# Alterations in Lipid and Inositol Metabolisms in Two Dopaminergic Disorders

**DOI:** 10.1371/journal.pone.0147129

**Published:** 2016-01-25

**Authors:** Eva C. Schulte, Elisabeth Altmaier, Hannah S. Berger, Kieu Trinh Do, Gabi Kastenmüller, Simone Wahl, Jerzy Adamski, Annette Peters, Jan Krumsiek, Karsten Suhre, Bernhard Haslinger, Andres Ceballos-Baumann, Christian Gieger, Juliane Winkelmann

**Affiliations:** 1 Neurologische Klinik und Poliklinik, Klinikum rechts der Isar, Technische Universität München, 81675, Munich, Germany; 2 Institut für Humangenetik, Helmholtz Zentrum München, 85764, Neuherberg, Germany; 3 Institute of Genetic Epidemiology, Helmholtz Zentrum München, 85764, Neuherberg, Germany; 4 Research Unit of Molecular Epidemiology, Helmholtz Zentrum München, 85764, Neuherberg, Germany; 5 Institute of Computational Biology, Helmholtz Zentrum München, 85764, Neuherberg, Germany; 6 Institute of Bioinformatics and Systems Biology, Helmholtz Zentrum München, 85764, Neuherberg, Germany; 7 Institute of Epidemiology II, Helmholtz Zentrum München, 85764, Neuherberg, Germany; 8 Institute of Experimental Genetics, Genome Analysis Center, Helmholtz Zentrum München, 85764, Neuherberg, Germany; 9 Lehrstuhl für Experimentelle Genetik, Technische Universität München, Freising-Weihenstephan, Germany; 10 Department of Physiology and Biophysics, Weill Cornell Medical College in Qatar, Qatar Foundation–Education City, PO Box 24144, Doha, Qatar; 11 Schön Klinik München Schwabing, Munich, Germany; 12 Institut für Humangenetik, Technische Universität München, 81675, Munich, Germany; 13 Munich Cluster for Systems Neurology (SyNergy), Munich, Germany; 14 Department of Neurology and Neurosciences, Stanford University, Palo Alto, CA, 94304, United States of America; University of Ulm, GERMANY

## Abstract

**Background:**

Serum metabolite profiling can be used to identify pathways involved in the pathogenesis of and potential biomarkers for a given disease. Both restless legs syndrome (RLS) and Parkinson`s disease (PD) represent movement disorders for which currently no blood-based biomarkers are available and whose pathogenesis has not been uncovered conclusively. We performed unbiased serum metabolite profiling in search of signature metabolic changes for both diseases.

**Methods:**

456 metabolites were quantified in serum samples of 1272 general population controls belonging to the KORA cohort, 82 PD cases and 95 RLS cases by liquid-phase chromatography and gas chromatography separation coupled with tandem mass spectrometry. Genetically determined metabotypes were calculated using genome-wide genotyping data for the 1272 general population controls.

**Results:**

After stringent quality control, we identified decreased levels of long-chain (polyunsaturated) fatty acids of individuals with PD compared to both RLS (PD vs. RLS: p = 0.0001 to 5.80x10^-9^) and general population controls (PD vs. KORA: p = 6.09x10^-5^ to 3.45x10^-32^). In RLS, inositol metabolites were increased specifically (RLS vs. KORA: p = 1.35x10^-6^ to 3.96x10^-7^). The impact of dopaminergic drugs was reflected in changes in the phenylalanine/tyrosine/dopamine metabolism observed in both individuals with RLS and PD.

**Conclusions:**

A first discovery approach using serum metabolite profiling in two dopamine-related movement disorders compared to a large general population sample identified significant alterations in the polyunsaturated fatty acid metabolism in PD and implicated the inositol metabolism in RLS. These results provide a starting point for further studies investigating new perspectives on factors involved in the pathogenesis of the two diseases as well as possible points of therapeutic intervention.

## Introduction

Serum metabolite profiling can be used to investigate pathways involved in disease pathogenesis and to identify potential biomarkers for a given disease. Such markers harbor great potential to empower patient care by aiding in (pre-)clinical diagnosis, monitoring of therapeutic responses, providing individualized therapy and improving the design of clinical trials, among others. [1] In the context of neurodegenerative conditions, a recent longitudinal study on mild cognitive impairment and Alzheimer`s disease (AD) illustrated that a panel of ten plasma lipids can be used to detect preclinical AD in cognitively normal older adults with 90% accuracy. [2] Both restless legs syndrome (RLS)—characterized by nightly limb dysaesthesias induced by rest or inactivity—and Parkinson`s disease (PD) represent common (prevalence in adult European populations between 1 and 10% [3, 4]) movement disorders for which currently no serological markers facilitating diagnosis or therapeutic management are available.

Although the mechanisms of action are likely not the same with different dopaminergic pathways involved, common to both diseases is a therapeutic response to L-Dopa and dopamine agonists and the near-ubiquitous use of these drugs by individuals with either disease. Accordingly, we decided to jointly evaluate both diseases to better differentiate secondary effects stemming from drug use from primary disease-relevant effects.

## Methods

### Study Samples

The Cooperative Health Research in the Region of Augsburg (KORA) study comprises a series of independent population-based epidemiological surveys and follow-up studies of participants living in Southern Germany [5]. The present study includes data from 1272 individuals (64.1±5.5 years, 47.7% female) belonging to the KORA S4 survey.

The PD sample consists of 82 cases (70.0±8.7 years, 50.0% female) diagnosed in accordance with the UK Brain Bank Criteria [6] at Schön Klinik München-Schwabing, specializing in PD, and Neurologische Klinik und Poliklinik, Klinikum rechts der Isar, Technische Universität München, both Munich, Germany.

In the 95 RLS cases (60.6±17.0 yrs; 70.5% female), diagnosis was based on the diagnostic criteria of the International RLS Study Group [7] as assessed in a personal interview. All RLS cases were recruited at the RLS outpatient clinic at Neurologische Klinik und Poliklinik, Klinikum rechts der Isar, Technische Universität München, Munich, Germany. In keeping with the previous GWAS [8, 9], we excluded individuals with secondary RLS due to uremia, dialysis, or anemia due to iron deficiency. Both individuals with RLS and individuals with PD were continued on their regular therapeutic regimes.

Participants’ written informed consent was obtained prior to the initiation of the study. The institutional review board of the Technische Universität München approved the study.

### Sample Collection

Sample collection was performed as described previously. [10, 11] Briefly, fasting serum was collected between 8 and 10 am into serum separator gel (controls) or plain serum (cases) tubes (both Sarstedt S-Monovette^®^, Sarstedt, Nümbrecht, Germany), left to coagulate for 30 to 60 minutes, centrifuged at 2500g and 10°C for 15 minutes and stored at -80°C until the time of metabolomic analysis.

### Metabolome Profiling

In each serum sample, 456 metabolites were profiled by Metabolon, Inc., Durham, NC, USA, using liquid-phase chromatography and gas chromatography separation coupled with tandem mass spectrometry. This analytical platform performs high-throughput, semi-quantitative analysis of analytical data to detect a broad spectrum of molecules with a high degree of confidence. [12] The added standards are not used for the calculation of the metabolite concentrations but to determine retention time. Relative intensity is measured sensitive to fluctuations caused by column change as well as instrument parameters. Fluctuations are day-dependent and run day normalization of the metabolic data was carried out: For each individual and each metabolite, the data were first divided by the day median of the respective metabolite and then multiplied with the overall median of this metabolite. Metabolite concentrations were log-transformed.

281 (61.6%) of the metabolites were chemically identified and include amino acids, acylcarnitines, carbohydrates, lipids, glycerophospholipids, small peptides, cofactors and vitamins, nucleotides and xenobiotics. For 175 (38.4%) additional metabolites, mass, MS/MS fragmentation and chromatographic retention time were also identified, but their biochemical identity remains unknown. Further details on these metabolites can be found in S1 Table. All samples were measured in a single batch.

### Network Analysis

Prior to Gaussian Graphical Models (GGMs) estimation, metabolite concentrations were log-transformed and standardized. Moreover, metabolites and samples with more than 20% and 10% missing values, respectively, were excluded from the dataset to preserve statistical power during partial correlation analysis. The remaining missing values were imputed with the MICE R package. We used the publicly available R package ‘GeneNet’, a shrinkage estimator-based approach for partial correlation calculation even in low-powered scenarios. Partial correlations were calculated correcting for the standard confounders age and sex, as well as RLS and PD. We defined edges between each set of two metabolites if both their Pearson correlation and their partial correlation were statistically significant with Bonferroni corrected α = 0.05. This avoids statistical artifacts occurring when Pearson correlations are zero and partial correlations are negative.

### Genome-Wide Genotyping

For all KORA individuals, genome-wide SNP data were already available. [10, 11, 13, 14] KORA S4/F4 samples were genotyped on the Affymetrix^®^ Axiom platform. Genotypes were called with the Affymetrix^®^ software and annotated to NCBI build 37. We excluded SNPs with call rates <98%, HWE p-values <5x10^-6^, and minor allele frequency <1%. SHAPEIT v2 was used for pre-phasing, imputation performed with IMPUTE v2.3.0 using the 1000G phase1 reference panel. In total,18,185,628 SNPs genotyped on the Affymetrix^®^ Axiom platform were available for analysis.

### Data Analysis

Our focus in the statistical analysis was to find differences between the healthy controls and the respective disease group. Thus, for the identification of metabolites associated with RLS and PD, respectively, a linear regression model with age and sex as confounders was used to model each metabolite concentration. Results were Bonferroni corrected and all p-values <1.09 x 10^−4^ (0.05/456 metabolites) were considered metabolome-wide significant. In addition, a comparison between RLS and PD was conducted using the same linear regression model.

In our study design, we are unable to preclude systematic differences in metabolites that showed differences from controls with the same direction of effect in both individuals with RLS and individuals with PD. Therefore, we focused our analyses on metabolites, which showed differences in either only individuals with PD or only individuals with RLS compared to controls.

Since ratios of metabolites were found to serve as proxies for enzymatic activity [13, 15], the ratios of the concentrations were calculated for all possible pairs of metabolites and the same linear regression analysis was conducted.

For the metabogenomic analysis aiming to identify associations between disease-associated SNPs and metabolite concentrations in the general population (KORA), a regression test with BMI, age, gender, HDL cholesterol, LDL cholesterol, total cholesterol, triglycerides, hypertension and diabetes as co-factors was applied for each SNP. The results were Bonferroni corrected (significance threshold of p<3.43x10^-6^ (0.05/32 candidate SNPs/456 metabolites)).

Network inference on the metabolomics data was performed by estimating Gaussian Graphical Models (GGMs) which are based on conditional dependencies (partial correlation coefficients) in multivariate Gaussian distributions. [14, 16, 17] For both RLS and PD, the regression coefficients and the logged-transformed p-values obtained from this regression analysis were mapped onto the filtered (Bonferroni-corrected) GGM, which was then visualized as node pie charts in Cytoscape with node coloring according to the direction (regression coefficients) of phenotypic association.

## Results

### Single Metabolites and Pathways Common to PD and RLS

**Fig 1:**
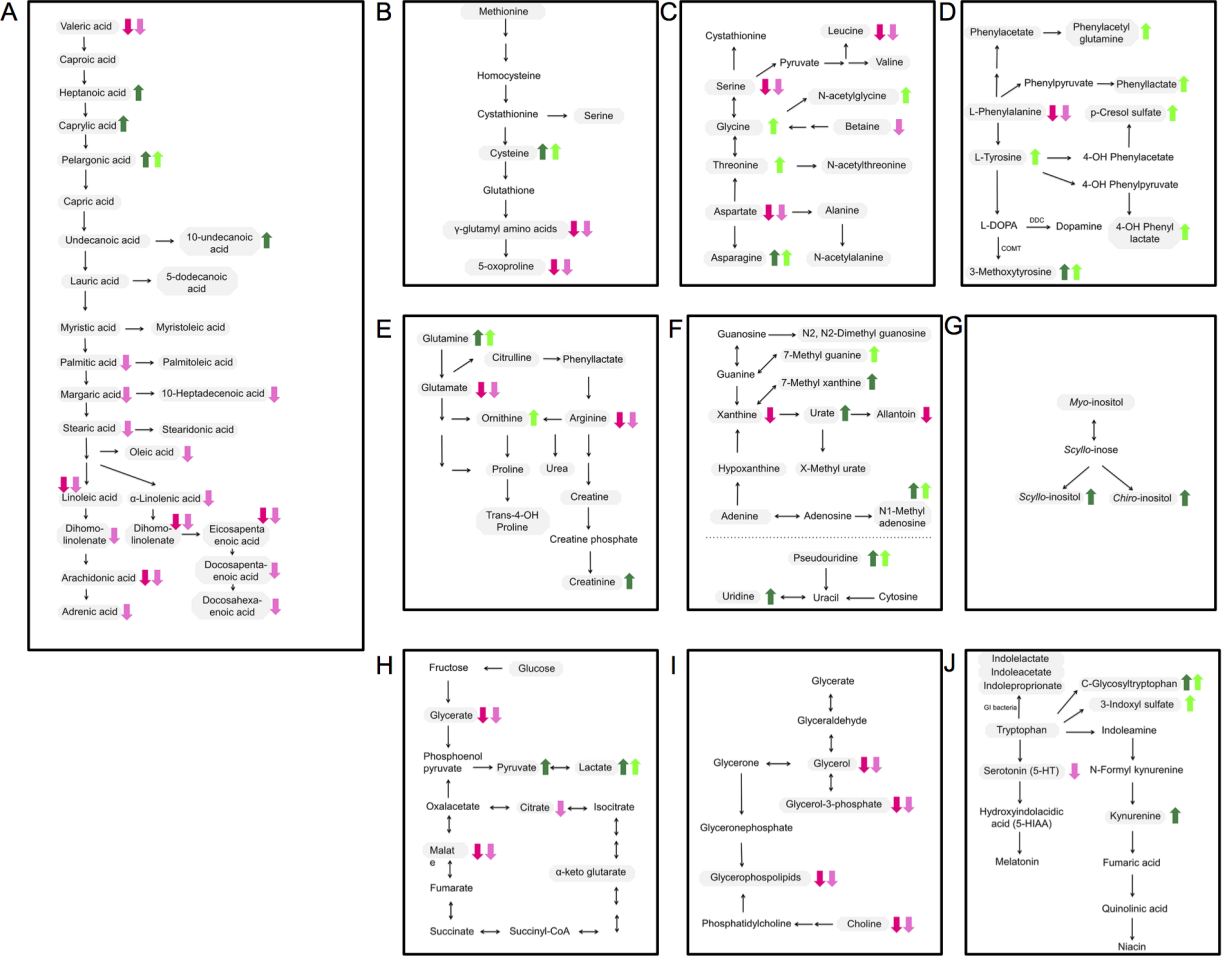
Changes in serum metabolite profiles in individuals with RLS (dark-colored arrows) and PD (light-colored arrows). Simplified representation of biochemical pathways according to the Kyoto Encyclopedia of Genes and Genomes (KEGG) (A) Fatty acid metabolism, (B) cysteine metabolism, (C) serine/glycine/threonine/aspartate/alanine metabolism, (D) phenylanalnine/tyrosine/L-DOPA metabolism, (E) glutamate metabolism and urea cycle, (F) purine and pyrimidine metabolism, (G) inositol metabolism, (H) citric acid cycle, (I) glycerol and choline metabolism, (J) tryptophan metabolism. Gray shading indicates metabolites measured, known metabolites in the employed metabolome panel.

In total, we identified 128 metabolites showing significantly different serum levels in individuals with PD compared to KORA controls while 106 metabolites differed significantly between individuals with RLS and controls (S1 Table). 37 metabolites were significantly altered between RLS and PD while 69 metabolites—among these 14 unidentified metabolites—showed significant unidirectional differences in both PD and RLS when compared to controls. For these metabolites, we could not exclude that these represent either drug-dependent effects or systematic false positive results. Among these metabolites (S1 Table) are a number of factors involved in the metabolism of fibrinogen and hemoglobin as well as polypeptide degradation products, which are likely the result of different serum tubes utilized to collect cases and controls. However, we also observed several changes in the phenylalanine/tyrosine/dopamine pathway expected to occur with dopaminergic treatment: attenuated levels of serum phenylalanine (↓: RLS vs. KORA, p = 1.40x10^-42^; PD vs. KORA, p = 4.10x10^-30^) and elevated levels of L-DOPA metabolite 3-methoxytyrosine reflecting the different doses of L-DOPA used in treating the two diseases (↑: RLS vs. KORA, p<1.37x10^-21^; PD vs. KORA, p = 1.04x10^-233^) (Fig 1D and S1 Table). Additionally, serum concentrations of threonate, the degradation product of antioxidant and tyrosine metabolism co-enzyme ascorbate/vitamin C, were also significantly increased in individuals with RLS as well as individuals with PD (RLS vs. KORA, p = 7.01x10^-235^; PD vs. KORA, p = 7.57x10^-144^).

Moreover, highly significant differences between cases and controls were also present in the homocystein/glutathione pathway (cysteine ↑: RLS vs. KORA, p<1.0x10^-300^; PD vs. KORA, p = 1.11x10^-298^; 5-oxoproline ↓: RLS vs. KORA, p = 1.40x10^-55^; PD vs. KORA, p = 2.44x10^-23^; gamma-glutamylvaline↓: RLS vs. KORA, p = 2.59x10^-11^; PD vs. KORA, p = 5.94x10^-16^; gamma-glutamylleucine ↓: RLS vs. KORA, p = 6.64x10^-9^; PD vs. KORA, p = 4.04x10^-12^, without metabolome-wide significant changes in methionine, 2-hydroxybutyrate or other gamma-glutamyl amino acids (S1 Table); Fig 1B).

Lastly, a number of alterations common to both RLS and PD cases compared to the general population were seen in amino acid, nucleotide, carnitine and glycero(phospho)lipid pathways (Fig 1A–1J, S1 Fig, S1 Table).

### Single Metabolites and Pathways Unique to Either PD or RLS

**Fig 2:**
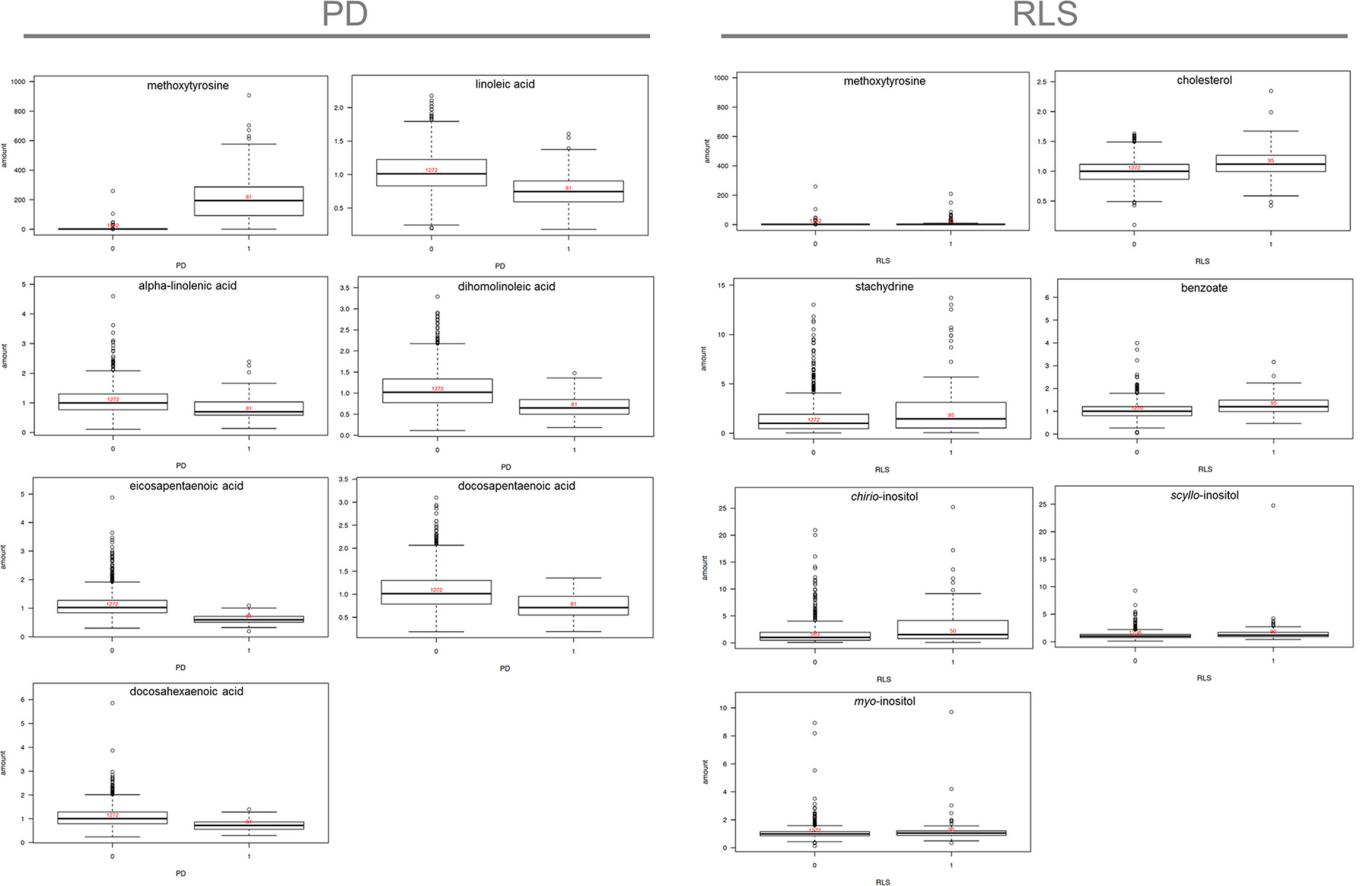
Selected box plots for metabolites of interest for PD (left) and RLS (right).

More importantly, we also observed a number of changes unique to either RLS or PD cases. While there were unidirectional changes in both in a number of components of the fatty acid metabolism (Fig 1A, Fig 2, S1 Table), in RLS these changes were characterized by an increase in medium chain fatty acids with 7 to 11 carbon atom backbones (RLS vs. KORA: p = 2.93x10^-6^ to 3.88x10^-19^) while in PD a decrease in long chain fatty acids and especially polyunsaturated fatty acids (PUFAs) with 16 to 22 carbon atom backbones was most pronounced (Table 1, Fig 1A). A total of 9 PUFAs were significantly attenuated in the serum of PD cases in comparison to RLS cases with p-values ranging from 0.0001 to 5.80x10^-9^ while 14 PUFAs with p-values ranging from 6.09x10^-5^ to 3.45x10^-32^ were decreased in comparison to KORA (Table 1, Fig 2).

**Table 1:** Generalized attenuation of polyunsaturated fatty acids (PUFAs) in individuals with PD compared to both individuals with RLS and the general population.

Metabolite	RLS		PD		RLS vs. PD
	direction of effect	p-value	direction of effect	p-value	p-value
valeric acid	↓	1.60x10^-8^	↓	7.47x10^-9^	0.29
caproic acid	↑	0.95	↓	0.26	0.19
heptanoic acid	↑	8.73x10^-16^	↑	0.0007	0.02
caprylic acid	↑	1.67x10^-5^	↑	0.006	0.25
pelargonic acid	↑	3.88x10^-19^	↑	1.57x10^-5^	0.04
capric acid	↑	0.02	↑	0.08	0.37
undecanoic acid	↑	2.93x10^-6^	↑	0.27	0.38
10-undecanoic acid	↑	0.01	↑	0.42	0.37
lauric acid	↑	0.01	↑	0.07	0.37
5-dodecanoic acid	↓	0.26	↓	0.51	0.22
myristic acid	↓	0.03	↓	0.005	0.21
myristoleic acid	↓	0.07	↓	0.2	0.50
palmitic acid	↓	0.05	↓	6.7x10^-8^	0.002
palmitoleic acid	↓	0.08	↓	0.004	0.11
margaric acid	↓	0.01	↓	1.46x10^-6^	0.01
10-heptadecenoic acid	↓	0.001	↓	6.09x10^-5^	0.15
stearic acid	↓	0.07	↓	3.99x10^-8^	0.0001
staridonic acid	↓	0.47	↓	0.02	7.63x10^-8^
oleic acid	↓	0.49	↓	5.95x10^-5^	0.007
linoleic acid	↓	3.73x10^-5^	↓	3.36x10^-16^	0.0001
α-linoleic acid	↓	0.53	↓	1.99x10^-6^	1.73x10^-5^
dihomolinoleic acid (20:3n3)	↓	0.0001	↓	2.07x10^-14^	4.15x10^-5^
dihomolinoleic acid (20:3n3 and n6)	↓	5.94x10^-18^	↓	3.45x10^-32^	0.0001
eicosapentaenoic acid	↓	5.12x10^-10^	↓	4.98x10^-21^	5.55x10^-7^
docosapentaenoic acid	↓	0.02	↓	1.38x10^-12^	5.76x10^-7^
docosahexaenoic acid	↓	0.17	↓	3.88x10^-13^	5.80x10^-9^
arachidonic acid	↓	6.85x10^-19^	↓	1.37x10^-13^	0.61
adrenic acid	↓	0.01	↓	5.23x10^-5^	0.07

Changes in fatty acid metabolism in the serum individuals with RLS (n = 95) and PD (n = 82) compared to the KORA general population cohort (n = 1272). Gray shading indicates p-values which remain significant after correction for multiple testing (Bonferroni).

Also, levels of serum cholesterol were found to be elevated in individuals with RLS compare to the general population (RLS vs. KORA, p = 4.73x10^-7^; PD vs. KORA, p = 0.15).

Differences were, moreover, seen in individuals with RLS in serum metabolites that may reflect renal dysfunction, although all RLS subjects included in the study had normal renal function parameters by clinical laboratory standards (data not shown). Compared to the general population, RLS cases showed increased serum creatinine (RLS vs. KORA, p = 1.09x10^-4^; PD vs. KORA, p = 0.03; S1 Table) and uric acid levels (RLS vs. KORA, p = 9.12x10^-7^; PD vs. KORA, p = 0.39; RLS vs. PD, p = 0.0005; Fig 1F, S1 Table). Protein-bound uremic toxin 4-ethyl-phenyl-sulfate was present in 1.9-fold higher concentration in individuals with RLS (RLS vs. KORA, p = 2.44x10^-8^; PD vs. KORA, p = 0.04). Possibly also in this context, two isomers of inositol—*scyllo*-inositol (RLS vs. KORA, p = 3.96x10^-7^; PD vs. KORA, p = 0.18; RLS vs. PD, p = 0.0002) and *chiro*-inositol (RLS vs. KORA, p = 1.35x10^-6^; PD vs. KORA, p = 0.95; RLS vs. PD, p = 0.02; only 46.8% of all samples measured (S1 Table))—but not the more common primary isomer *myo*-inositol (RLS vs. KORA, p = 0.00025; PD vs. KORA, p = 0.16; RLS vs. PD, p = 0.001) were specifically increased in individuals with RLS.

Lastly, a number of substances found in dietary and herbal medicinal products were increased in individuals with RLS compared to KORA. These include thymol sulfate (RLS vs. KORA, p = 3.38x10^-5^; PD vs. KORA, p = 0.76) used as a fungizide and component of toothpastes and mouth waters, stachydrine/proline betaine (RLS vs. KORA, p = 6.00x10^-9^; PD vs. KORA, p = 0.88) found in citrus fruits and herbal medicines, and benzoate (RLS vs. KORA, p = 6.46x10^-10^; PD vs. KORA, p = 0.002), a component of foods such as berries and dairy products which is also used as a preservative for food and tobacco (Fig 2).

In individuals with PD, the most uniquely changed metabolites were identified among the unknown substances. Here, 24 metabolites with p-values ranging from 8.64x10^-5^ to 1.22x10^-24^ were altered only in PD but not RLS cases when compared with the general population.

### Ratios and Profiles

Both a ratio-based evaluation of metabolite concentrations and a feature selection approach did not yield additional information compared to the single-metabolite analysis in either PD or RLS (data not shown). Methoxytyrosine levels near completely differentiated PD cases from controls (p<2.0x10^-16^) but are likely the result of dopaminergic medication (Fig 2).

### Metabolomics Meets Genetics

It has been demonstrated that common genetic variants can show large effects at the metabolite level. [10] Yet, we did not see metabolite changes specifically associated with any of 32 common variants known to be associated with increased risk for either PD [18, 19] or RLS [8, 9, 20] (S2 Table) in the 1272 KORA individuals.

## Discussion

Serum metabolite profiling provides a relatively novel tool to query an easily accessible bio specimen (i.e. blood) for reflections of pathophysiologic processes and potential biomarkers in an unbiased fashion. In our study, we examined serum metabolite profiles in individuals with RLS and individuals with PD and compared them to a large number of KORA general population controls. One strength of our study is the fact that all samples were measured together in the same batch, thus precluding batch effects. Also, comparison to a large set of controls invested the study with the power necessary to detect smaller scale effects, which may have contributed to the identification of changes in entire pathways rather than singular metabolites. With regard to RLS, to our knowledge, this study represents the first investigation of serum metabolomics. Although at least with regard to serum drug and protein levels no clinically significant alterations were seen between serum gel and standard serum tubes [21], a weakness, nonetheless, of our study is the use of different serum tubes to collect case and control samples and the subsequent possible introduction of systematic errors, which we tried to account for in our analysis. Moreover, linear regression analysis was corrected for age, sex and drug use, of which especially age and sex are known to influence serum metabolite profiles. [22, 23] Body mass index (BMI) represents another factor influencing serum metabolite profiles [22], however, no association between BMI and either PD or RLS has been reported, to date. With the exception of uric acid (p_corrected_ = 3.71x10^-26^; lin reg), none of the metabolites implicated in either PD or RLS were significantly altered by BMI in the KORA survey (data not shown), suggesting that the observed changes are, for the most part, independent of age and BMI.

With regard to PD, our data recapitulate observed changes in glutamate [24] and leucine [24] pathways though not uniformly in the same reported direction of effect and for the same metabolites.

Moreover, we were also able to substantiate a large prospective study that described higher serum cholesterol levels associated with an increased risk for RLS [25], strengthening the validity of the observed metabolite changes.

Some of the most prominent alterations common to both RLS and PD samples occurred in the phenylalanine/tyrosine/L-DOPA pathway and are likely related to the intake to dopaminergic medication in both groups. Homocysteinaemia alongside elevated serum cysteine concentrations is known to be related to L-DOPA intake [26] and is also reflected in our data. However, glutathione, a direct metabolite of cysteine, which was not measured in our samples, has also been shown to be elevated in unmedicated PD patients compared to controls [27], suggesting a possible dysfunction in this pathway independent of drug effects. However, since KORA represents the population at large the number of individuals using dopaminergic drugs was too small for a deeper analysis.

In PD, the most pronounced changes were observed with regard to PUFAs. A small study of 20 de novo PD patients and 20 healthy controls also saw attenuated levels of C16 to C18 saturated and unsaturated FAs in the plasma of the PD cases. [24] This finding is especially interesting in light of the fact that PUFAs are ascribed neuroprotective properties. [28] For example, in a longitudinal nutritional study of the general population, it was observed that decreased PUFA consumption was related to an increased risk for PD. [29] Further, dietary supplementation with both a mixture of essential PUFAs [30] or ethyl-eicosapentaenoate [31] or docosahexaenoic acid (DHA) [28, 32], which we also found to be decreased in the serum of PD patients, has been shown to protect dopaminergic neurons against cell death in mouse and rat MPTP-models of PD. Also, α-synuclein interacts directly with PUFAs [33, 34] and mice harboring the p.A53T variant of α-synuclein (*Snca*) exhibit increased α-synuclein oligomerization when fed a DHA-rich diet. [35] Lastly, PUFA levels were also reduced in the Parkinsonian substantia nigra when compared to both other Parkinsonian brain regions and to control tissue. [36] Taken together, these results may reflect an increased level of lipid peroxidation and oxidative stress in CNS tissues of relevance to PD [37] or could be indicative of special dietary habits or malresorption of essential fatty acids in individuals with PD.

*Scyllo*- and *chiro-*inositol represent two of the most specifically altered metabolites in RLS. Inositols and especially *myo*-inositol are synthesized in the kidney and also play a role in renal osmoregulation. [38] *Myo*-inositol oxygenase has been proposed as a clinical marker of acute kidney failure. [38] Inositols, moreover, serve as principal components of lipid membranes and secondary messengers in Ca^++^ signaling. Little is known on the biological function of the less common inositols. Yet, *scyllo*- and *chiro*-inositols have been implicated as potential therapeutic agents in a number of neuropsychiatric diseases ranging from Alzheimer`s [39] to obsessive compulsive disorder [40]. Although the serum inositol alterations in RLS were highly specific, it is unclear whether they could reflect changes in renal function, neuronal membrane stability, or a yet unknown pathophysiologic process pertinent to RLS. While it is well established that renal insufficiency is one of the major causes of secondary RLS, so far, there has not been any indication of impaired renal function in individuals with primary RLS. This result might indicate that even minimal changes in renal function could be implicated in triggering symptoms of RLS in individuals with a (genetic) predisposition, possibly rendering the border between primary and secondary RLS less well-defined than previously assumed.

In summary, serum metabolite profiling in two dopamine-related movement disorders compared to a large general population sample identified significant alterations in the PUFA metabolism in PD and implicated the inositol metabolism as well as three xenobiotics of unknown relevance in RLS. Our study is a first discovery analysis especially in the field of RLS basing on strict Bonferroni thresholds. Thus, a replication analysis as well as clinical or functional studies have to be conducted to be able to make clear conclusions. Although our study is compromised by the fact that it lacks longitudinal observation, making conclusions regarding primary vs. secondary pathophysiologic changes very difficult, it, nonetheless, provides new perspectives on factors potentially involved in bringing about the two diseases as well as possible points of therapeutic intervention. In our study, we were unable to identify a single metabolite or metabolite profile that could fully differentiate individuals with either disease from the general population as well as from each other. Although this remains the ultimate goal, it is possible that a single method will not be sufficient to identify the ideal biomarker for either disease and that a combination of approaches might be necessary to achieve this goal.

## Supporting Information

S1 Fig Metabolic pathway network analysis. Changes in PD are represented on the right side, in RLS on the left side of each node; red coloring indicates a decrease in the given metabolite while green coloring indicates an increase.
(PDF)

S1 Table Measurements and statistical analysis for 456 metabolites characterized in the serum of individuals with RLS, PD and the general population. (DOCX)

S2 Table Single nucleotide polymorphisms (SNPs) used in metabogenomics analysis. (DOCX)
