# Supplementary figures and images for: Alterations in Lipid and Inositol Metabolisms in Two Dopaminergic Disorders
Source: PLoS One. 2016 Jan 25;11(1):e0147129. doi: 10.1371/journal.pone.0147129 (PMC4726488; doi:10.1371/journal.pone.0147129)

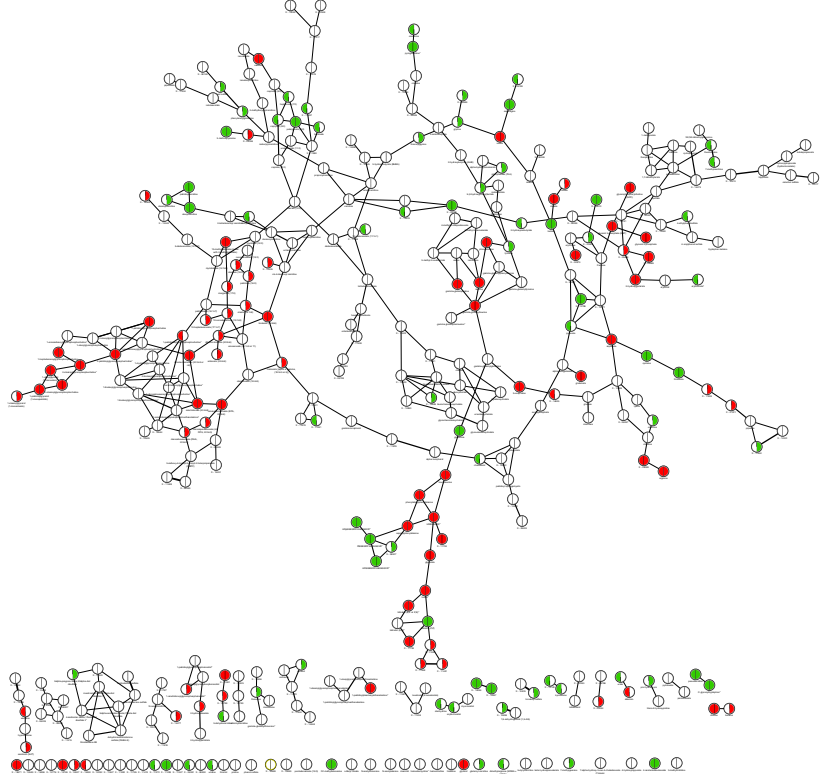

Supplement: S1 Fig — Changes in PD are represented on the right side, in RLS on the left side of each node; red coloring indicates a decrease in the given metabolite while green coloring indicates an increase. (PDF) [file pone.0147129.s001.pdf]
