# Supplementary material for: Alterations in Lipid and Inositol Metabolisms in Two Dopaminergic Disorders
Source: PLoS One. 2016 Jan 25;11(1):e0147129. doi: 10.1371/journal.pone.0147129 (PMC4726488; doi:10.1371/journal.pone.0147129)
Supplement: S1 Table — (DOCX) [file pone.0147129.s002.docx]

|  | |  |  |  |  |  |  |  |  |  |  |  |  |  |  |  |  |  |  |
| --- | --- | --- | --- | --- | --- | --- | --- | --- | --- | --- | --- | --- | --- | --- | --- | --- | --- | --- | --- |
|  |  |  |  |  |  |  |  |  |  |  |  |  |  |  |  |  |  |  |  |
| **Metabolite ID** | **Metabolite** | **Status** | **Pathway** | **Super-pathway** | **KORA** | | |  | **PD** | | |  |  | **RLS** | | |  |  | **RLS vs. PD** |
|  |  |  |  |  | **N** | **Mean** | **SE** |  | **N** | **Mean** | **SE** | **p-value** |  | **N** | **Mean** | **SE** | **p-value** |  | **p-value** |
| M32339 | alanine | known | Alanine and aspartate metabolism | Amino acid | 1272 | 1.029 | 0.106 |  | 81 | 1.008 | 0.029 | 0.114197568 |  | 95 | 1.042 | 0.027 | 0.539291108 |  | 0.405558143 |
| M34283 | asparagine | known | Alanine and aspartate metabolism | Amino acid | 1272 | 1.006 | 0.110 |  | 81 | 1.206 | 0.028 | 4.31E-13 |  | 95 | 1.163 | 0.026 | 4.28E-10 |  | 0.272859687 |
| M15996 | aspartate | known | Alanine and aspartate metabolism | Amino acid | 1272 | 1.122 | 0.216 |  | 81 | 0.475 | 0.054 | 5.32E-31 |  | 95 | 0.345 | 0.049 | 5.51E-52 |  | 9.73E-05 |
| M01585 | N-acetylalanine | known | Alanine and aspartate metabolism | Amino acid | 1271 | 1.015 | 0.080 |  | 81 | 1.140 | 0.026 | 0.00172292 |  | 95 | 1.043 | 0.024 | 0.103932182 |  | 1 |
| M32348 | erythronate* | known | Aminosugars metabolism | Carbohydrate | 1272 | 1.044 | 0.107 |  | 81 | 1.220 | 0.047 | 0.102678684 |  | 95 | 1.246 | 0.050 | 2.10E-06 |  | 0.444886727 |
| M27718 | threonate | known | Ascorbate and aldarate metabolism | Cofactors and vitamins | 1272 | 0.982 | 0.210 |  | 81 | 2.956 | 0.068 | 7.57E-144 |  | 95 | 4.224 | 0.079 | 7.01E-235 |  | 0.616397662 |
| M00513 | 2-hydroxyhippurate (salicylurate) | known | Benzoate metabolism | Xenobiotics | 677 | 11.176 | 0.092 |  | 33 | 18.082 | 7.392 | 0.768786171 |  | 51 | 23.158 | 6.354 | 0.052113043 |  | 0.144661989 |
| M21044 | 4-ethylphenylsulfate | known | Benzoate metabolism | Xenobiotics | 1079 | 1.509 | 0.173 |  | 55 | 1.034 | 0.283 | 0.04866753 |  | 80 | 3.377 | 0.335 | 2.44E-08 |  | 0.002920151 |
| M31453 | 4-hydroxyhippurate | known | Benzoate metabolism | Xenobiotics | 1132 | 1.221 | 0.134 |  | 78 | 1.397 | 0.143 | 0.551762001 |  | 88 | 1.508 | 0.134 | 0.025505317 |  | 0.546286821 |
| M31454 | 4-vinylphenol sulfate | known | Benzoate metabolism | Xenobiotics | 1259 | 1.596 | 0.232 |  | 74 | 1.115 | 0.201 | 0.115840941 |  | 94 | 1.566 | 0.174 | 0.382981681 |  | 7.58E-06 |
| M01302 | benzoate | known | Benzoate metabolism | Xenobiotics | 1270 | 1.037 | 0.063 |  | 81 | 1.165 | 0.044 | 0.002324118 |  | 95 | 1.284 | 0.038 | 6.46E-10 |  | 0.060182306 |
| M32322 | catechol sulfate | known | Benzoate metabolism | Xenobiotics | 1267 | 1.120 | 0.178 |  | 81 | 1.046 | 0.075 | 0.361038212 |  | 95 | 1.332 | 0.071 | 0.005480136 |  | 0.006610197 |
| M00053 | hippurate | known | Benzoate metabolism | Xenobiotics | 1272 | 1.292 | 0.051 |  | 81 | 1.297 | 0.141 | 0.592775183 |  | 95 | 1.456 | 0.131 | 0.229495593 |  | 0.887682218 |
| M32672 | cholate | known | Bile acid metabolism | Lipid | 1191 | 2.970 | 0.255 |  | 74 | 3.716 | 0.785 | 0.99447607 |  | 92 | 2.315 | 0.664 | 0.652009254 |  | 0.546857153 |
| M01494 | deoxycholate | known | Bile acid metabolism | Lipid | 1031 | 1.371 | 0.070 |  | 67 | 2.045 | 0.202 | 0.008908937 |  | 74 | 1.031 | 0.156 | 0.073814768 |  | 0.003072852 |
| M35159 | glycochenodeoxycholate | known | Bile acid metabolism | Lipid | 1255 | 1.685 | 0.220 |  | 81 | 2.202 | 0.265 | 0.036403483 |  | 94 | 1.108 | 0.228 | 0.02910678 |  | 1.29E-05 |
| M03141 | glycocholate | known | Bile acid metabolism | Lipid | 984 | 1.571 | 0.116 |  | 61 | 1.769 | 0.287 | 0.261695242 |  | 70 | 1.076 | 0.245 | 0.040784657 |  | 0.002268921 |
| M32338 | glycoursodeoxycholate | known | Bile acid metabolism | Lipid | 1196 | 1.844 | 0.124 |  | 78 | 2.611 | 0.368 | 0.046494644 |  | 88 | 1.788 | 0.341 | 0.960113882 |  | 0.0129784 |
| M27710 | hyodeoxycholate | known | Bile acid metabolism | Lipid | 1111 | 1.574 | 0.242 |  | 70 | 2.336 | 0.254 | 0.008214732 |  | 82 | 2.216 | 0.232 | 0.002194901 |  | 0.320249841 |
| M33939 | taurochenodeoxycholate | known | Bile acid metabolism | Lipid | 1126 | 1.667 | 0.125 |  | 68 | 2.018 | 0.483 | 0.275723207 |  | 82 | 1.086 | 0.421 | 0.131910707 |  | 0.844890543 |
| M32315 | taurocholate | known | Bile acid metabolism | Lipid | 764 | 3.050 | 0.102 |  | 55 | 2.811 | 2.008 | 0.854493562 |  | 60 | 1.639 | 1.860 | 0.328584243 |  | 0.093390937 |
| M01284 | taurolithocholate 3-sulfate | known | Bile acid metabolism | Lipid | 1164 | 1.258 | 0.110 |  | 73 | 1.589 | 0.149 | 0.050513595 |  | 80 | 1.251 | 0.132 | 0.87052188 |  | 0.535212649 |
| M01558 | ursodeoxycholate | known | Bile acid metabolism | Lipid | 1113 | 1.496 | 0.105 |  | 77 | 2.593 | 0.245 | 0.000230619 |  | 83 | 1.684 | 0.221 | 0.206299891 |  | 0.000878115 |
| M15677 | 2-aminobutyrate | known | Butanoate metabolism | Amino acid | 1272 | 1.031 | 0.339 |  | 81 | 1.142 | 0.033 | 0.000306258 |  | 95 | 1.017 | 0.030 | 0.930304543 |  | 0.065952875 |
| M00059 | 2-tetradecenoyl carnitine | known | Carnitine metabolism | Lipid | 1256 | 1.206 | 0.058 |  | 80 | 1.374 | 0.104 | 0.301103 |  | 94 | 1.370 | 0.094 | 0.014698222 |  | 0.254946824 |
| M35439 | 3-dehydrocarnitine* | known | Carnitine metabolism | Lipid | 1272 | 1.039 | 0.146 |  | 81 | 1.226 | 0.044 | 0.002605631 |  | 95 | 1.267 | 0.041 | 7.72E-08 |  | 0.63539851 |
| M01301 | acetylcarnitine | known | Carnitine metabolism | Lipid | 1272 | 1.038 | 0.083 |  | 81 | 0.964 | 0.032 | 0.000280346 |  | 95 | 1.124 | 0.030 | 0.000549678 |  | 7.99E-07 |
| M01444 | carnitine | known | Carnitine metabolism | Lipid | 1272 | 1.000 | 0.177 |  | 81 | 1.042 | 0.018 | 0.004763904 |  | 95 | 0.983 | 0.016 | 0.285964033 |  | 0.009301699 |
| M35635 | cis-4-decenoyl carnitine | known | Carnitine metabolism | Lipid | 1272 | 1.144 | 0.241 |  | 81 | 1.051 | 0.085 | 0.022494058 |  | 95 | 1.090 | 0.076 | 0.912127177 |  | 0.037582686 |
| M32197 | decanoylcarnitine | known | Carnitine metabolism | Lipid | 1272 | 1.155 | 0.164 |  | 81 | 1.537 | 0.133 | 0.021623038 |  | 95 | 1.321 | 0.117 | 0.072798687 |  | 0.550863206 |
| M12017 | hexanoylcarnitine | known | Carnitine metabolism | Lipid | 1272 | 1.105 | 0.140 |  | 81 | 1.442 | 0.127 | 0.047997356 |  | 95 | 1.186 | 0.111 | 0.339685075 |  | 0.333610063 |
| M15749 | laurylcarnitine | known | Carnitine metabolism | Lipid | 1052 | 1.183 | 0.241 |  | 80 | 1.668 | 0.111 | 0.000174641 |  | 91 | 1.538 | 0.105 | 6.09E-05 |  | 0.379880408 |
| M00541 | octanoylcarnitine | known | Carnitine metabolism | Lipid | 1272 | 1.164 | 0.217 |  | 81 | 1.539 | 0.163 | 0.089632816 |  | 95 | 1.274 | 0.143 | 0.322226847 |  | 0.952078 |
| M36103 | oleoylcarnitine | known | Carnitine metabolism | Lipid | 1268 | 1.088 | 0.311 |  | 81 | 1.487 | 0.064 | 1.72E-08 |  | 95 | 1.347 | 0.058 | 7.30E-08 |  | 0.007109341 |
| M32553 | palmitoylcarnitine | known | Carnitine metabolism | Lipid | 1265 | 1.099 | 0.275 |  | 81 | 1.460 | 0.063 | 3.87E-08 |  | 95 | 1.294 | 0.056 | 1.67E-05 |  | 0.550574445 |
| M15958 | stearoylcarnitine | known | Carnitine metabolism | Lipid | 1221 | 1.086 | 0.167 |  | 79 | 1.396 | 0.060 | 6.23E-06 |  | 94 | 1.208 | 0.053 | 0.001061577 |  | 7.63E-08 |
| M35126 | glycerol 2-phosphate | known | Chemical | Xenobiotics | 1270 | 1.058 | 0.282 |  | 81 | 0.895 | 0.040 | 4.81E-05 |  | 92 | 0.881 | 0.037 | 9.92E-07 |  | 0.109475858 |
| M00064 | creatine | known | Creatine metabolism | Amino acid | 1272 | 1.104 | 0.055 |  | 81 | 0.936 | 0.057 | 0.013783058 |  | 95 | 1.074 | 0.053 | 0.009819794 |  | 0.593281916 |
| M22130 | creatinine | known | Creatine metabolism | Amino acid | 1272 | 1.009 | 0.141 |  | 81 | 1.074 | 0.025 | 0.038572731 |  | 95 | 1.058 | 0.023 | 0.000109403 |  | 0.651260781 |
| M01299 | 2-hydroxybutyrate (AHB) | known | Cysteine, methionine, SAM, taurine metabolism | Amino acid | 1272 | 1.108 | 0.077 |  | 81 | 0.997 | 0.061 | 0.088018506 |  | 95 | 1.065 | 0.055 | 0.720922202 |  | 0.123583137 |
| M27672 | cysteine | known | Cysteine, methionine, SAM, taurine metabolism | Amino acid | 1263 | 0.977 | 0.191 |  | 81 | 3.326 | 0.048 | 1.11E-298 |  | 95 | 3.663 | 0.049 | 0 |  | 0.197895373 |
| M32675 | methionine | known | Cysteine, methionine, SAM, taurine metabolism | Amino acid | 1268 | 1.005 | 0.090 |  | 81 | 1.050 | 0.020 | 0.009598945 |  | 95 | 1.016 | 0.018 | 0.124985661 |  | 0.011561899 |
| M27513 | aspartylphenylalanine | known | Dipeptide | Peptide | 1269 | 1.187 | 0.193 |  | 80 | 0.401 | 0.069 | 4.75E-30 |  | 89 | 0.255 | 0.064 | 2.53E-43 |  | 0.000143891 |
| M18349 | cyclo(leu-pro) | known | Dipeptide | Peptide | 1069 | 1.345 | 0.149 |  | 64 | 0.996 | 0.174 | 0.068506933 |  | 76 | 1.155 | 0.157 | 0.579268393 |  | 0.054961479 |
| M32405 | leucylleucine | known | Dipeptide | Peptide | 1250 | 1.132 | 0.218 |  | 65 | 0.523 | 0.065 | 5.47E-21 |  | 81 | 0.535 | 0.056 | 4.61E-25 |  | 0.906304779 |
| M15140 | phenylalanylphenylalanine | known | Dipeptide | Peptide | 1271 | 1.111 | 0.105 |  | 81 | 0.556 | 0.048 | 5.68E-28 |  | 95 | 0.491 | 0.043 | 4.00E-45 |  | 0.247279096 |
| M02342 | pro-hydroxy-pro | known | Dipeptide | Peptide | 1271 | 1.045 | 0.164 |  | 81 | 1.344 | 0.045 | 4.25E-08 |  | 95 | 1.077 | 0.041 | 0.353421968 |  | 0.006261233 |
| M00054 | 2-hydroxyacetaminophen sulfate* | known | Drug | Xenobiotics | 27 | 1.312 | 0.062 |  | 21 | 0.867 | 0.246 | 0.112568433 |  | 6 | 1.084 | 0.461 | 0.756495883 |  | 0.736041974 |
| M37097 | 2-methoxyacetaminophen sulfate* | known | Drug | Xenobiotics | 21 | 1.475 | 0.463 |  | NA | NA | NA | NA |  | 4 | 1.018 | 1.088 | 0.668178987 |  | 0.50489284 |
| M01638 | 3-(cystein-S-yl)acetaminophen* | known | Drug | Xenobiotics | 20 | 0.937 | 0.103 |  | NA | NA | NA | NA |  | 4 | 1.089 | 0.166 | 0.314530119 |  | 0.282365745 |
| M02132 | 4-acetamidophenol | known | Drug | Xenobiotics | 23 | 0.945 | 0.097 |  | 2 | 1.480 | 0.327 | 0.08511743 |  | 4 | 1.078 | 0.227 | 0.493416383 |  | 0.124991834 |
| M36808 | atenolol | known | Drug | Xenobiotics | 17 | 0.945 | 0.095 |  | 0 | NA | NA | NA |  | 0 | NA | NA | NA |  | 1 |
| M22138 | carbamazepine* | known | Drug | Xenobiotics | 9 | 1.000 | 0.170 |  | 0 | NA | NA | NA |  | 0 | NA | NA | NA |  | 1 |
| M15630 | ibuprofen | known | Drug | Xenobiotics | 10 | 0.949 | 0.226 |  | 5 | 0.955 | 0.214 | 0.841621253 |  | 8 | 2.432 | 1.430 | 0.588680391 |  | 0.002072804 |
| M01493 | metoprolol acid metabolite* | known | Drug | Xenobiotics | 116 | 1.139 | 0.119 |  | 5 | 1.258 | 0.462 | 0.977943229 |  | 7 | 1.895 | 0.404 | 0.104275801 |  | 0.367064878 |
| M01898 | naproxen | known | Drug | Xenobiotics | 3 | 1.000 | 0.111 |  | 0 | NA | NA | NA |  | 0 | NA | NA | NA |  | 0.973752437 |
| M32319 | p-acetamidophenylglucuronide | known | Drug | Xenobiotics | 38 | 0.967 | 0.199 |  | 2 | 1.341 | 0.322 | 0.228801496 |  | 4 | 1.148 | 0.232 | 0.472809568 |  | 0.0001172 |
| M01670 | salicylate | known | Drug | Xenobiotics | 53 | 4.400 | 0.129 |  | 5 | 0.992 | 7.624 | 0.764675532 |  | 7 | 1.911 | 6.071 | 0.628129609 |  | 0.060931221 |
| M22030 | salicyluric glucuronide* | known | Drug | Xenobiotics | 133 | 2.105 | 0.135 |  | 9 | 1.991 | 1.341 | 0.940891656 |  | 12 | 4.170 | 1.178 | 0.076077248 |  | 0.514200377 |
| M35431 | thromboxane B2 | known | Eicosanoid | Lipid | 1260 | 1.264 | 0.143 |  | 80 | 1.012 | 0.106 | 0.984684908 |  | 95 | 1.120 | 0.095 | 0.535033944 |  | 7.10E-05 |
| M32397 | dihomo-linolenate (20:3n3 or n6) | known | Essential fatty acid | Lipid | 1272 | 1.068 | 0.154 |  | 81 | 0.695 | 0.031 | 3.45E-32 |  | 95 | 0.823 | 0.028 | 5.94E-18 |  | 0.000121343 |
| M21047 | docosahexaenoate (DHA, 22:6n3) | known | Essential fatty acid | Lipid | 1272 | 1.085 | 0.092 |  | 81 | 0.735 | 0.050 | 3.88E-13 |  | 95 | 1.021 | 0.046 | 0.175089975 |  | 5.80E-09 |
| M15676 | docosapentaenoate (n3 DPA, 22:5n3) | known | Essential fatty acid | Lipid | 1272 | 1.080 | 0.111 |  | 81 | 0.741 | 0.049 | 1.38E-12 |  | 95 | 0.988 | 0.045 | 0.022083235 |  | 5.76E-07 |
| M22116 | eicosapentaenoate (EPA, 20:5n3) | known | Essential fatty acid | Lipid | 1272 | 1.111 | 0.114 |  | 81 | 0.618 | 0.051 | 4.98E-21 |  | 95 | 0.809 | 0.046 | 5.12E-10 |  | 5.55E-07 |
| M33937 | linoleate (18:2n6) | known | Essential fatty acid | Lipid | 1272 | 1.031 | 0.215 |  | 81 | 0.770 | 0.035 | 3.36E-16 |  | 95 | 0.915 | 0.032 | 3.73E-05 |  | 0.000821207 |
| M12129 | linolenate [alpha or gamma, (18:3n3 or 6)] | known | Essential fatty acid | Lipid | 1272 | 1.072 | 0.158 |  | 81 | 0.834 | 0.054 | 1.99E-06 |  | 95 | 1.130 | 0.049 | 0.531199147 |  | 0.000140115 |
| M35433 | isovalerate | known | Fatty acid metabolism | Lipid | 1262 | 1.077 | 0.194 |  | 81 | 1.121 | 0.045 | 0.424955744 |  | 95 | 0.908 | 0.040 | 0.001465672 |  | 0.592815463 |
| M33441 | butyrylcarnitine | known | Fatty acid metabolism (also BCAA metabolism) | Lipid | 1272 | 1.237 | 0.198 |  | 81 | 1.187 | 0.117 | 0.303677571 |  | 95 | 1.337 | 0.108 | 0.15732852 |  | 0.060511889 |
| M01125 | propionylcarnitine | known | Fatty acid metabolism (also BCAA metabolism) | Lipid | 1272 | 1.051 | 0.084 |  | 81 | 1.043 | 0.039 | 0.404818695 |  | 95 | 0.965 | 0.034 | 0.166410552 |  | 0.000317851 |
| M34407 | 15-methylpalmitate (isobar with 2-methylpalmitate) | known | Fatty acid, branched | Lipid | 1262 | 1.049 | 0.169 |  | 81 | 0.886 | 0.035 | 1.17E-07 |  | 94 | 0.946 | 0.032 | 2.87E-05 |  | 0.134102408 |
| M00060 | 2-hydroxyglutarate | known | Fatty acid, dicarboxylate | Lipid | 1250 | 1.224 | 0.069 |  | 79 | 0.794 | 0.084 | 6.75E-08 |  | 92 | 0.794 | 0.078 | 3.19E-08 |  | 0.626955261 |
| M22177 | 3-carboxy-4-methyl-5-propyl-2-furanpropanoate (CMPF) | known | Fatty acid, dicarboxylate | Lipid | 1272 | 1.292 | 0.179 |  | 81 | 1.085 | 0.136 | 0.050133861 |  | 95 | 1.152 | 0.119 | 0.566554716 |  | 0.006555021 |
| M35428 | dodecanedioate | known | Fatty acid, dicarboxylate | Lipid | 1211 | 1.081 | 0.155 |  | 77 | 1.128 | 0.058 | 0.964833721 |  | 86 | 1.153 | 0.056 | 0.14646381 |  | 0.224282437 |
| M01649 | hexadecanedioate | known | Fatty acid, dicarboxylate | Lipid | 1271 | 1.204 | 0.061 |  | 81 | 1.435 | 0.104 | 0.225607411 |  | 95 | 1.117 | 0.089 | 0.626694669 |  | 0.027561302 |
| M33477 | octadecanedioate | known | Fatty acid, dicarboxylate | Lipid | 1269 | 1.046 | 0.125 |  | 81 | 1.217 | 0.045 | 0.000820398 |  | 95 | 1.158 | 0.040 | 0.00036987 |  | 0.14443554 |
| M27722 | tetradecanedioate | known | Fatty acid, dicarboxylate | Lipid | 1267 | 1.241 | 0.203 |  | 81 | 1.329 | 0.108 | 0.985877081 |  | 94 | 1.228 | 0.098 | 0.881244051 |  | 0.624388164 |
| M00577 | n-Butyl Oleate | known | Fatty acid, ester | Lipid | 1026 | 1.042 | 0.230 |  | 57 | 1.059 | 0.050 | 0.878070655 |  | 79 | 1.060 | 0.043 | 0.735063891 |  | 0.658964603 |
| M15335 | 2-hydroxypalmitate | known | Fatty acid, monohydroxy | Lipid | 1272 | 1.030 | 0.429 |  | 81 | 0.955 | 0.023 | 0.000458712 |  | 95 | 0.943 | 0.021 | 0.000241911 |  | 0.931650441 |
| M00584 | 2-hydroxystearate | known | Fatty acid, monohydroxy | Lipid | 1272 | 1.046 | 0.122 |  | 81 | 0.918 | 0.029 | 2.29E-06 |  | 95 | 0.948 | 0.026 | 0.000207854 |  | 0.168947617 |
| M20675 | ADpSGEGDFXAEGGGVR* | known | Fibrinogen cleavage peptide | Peptide | 1019 | 1.135 | 0.165 |  | 77 | 10.765 | 0.283 | 7.82E-166 |  | 92 | 9.586 | 0.196 | 3.23E-240 |  | 0.566685008 |
| M21049 | ADSGEGDFXAEGGGVR* | known | Fibrinogen cleavage peptide | Peptide | 1270 | 1.027 | 0.204 |  | 81 | 4.178 | 0.102 | 4.43E-148 |  | 95 | 4.524 | 0.144 | 1.13E-109 |  | 0.063442532 |
| M20489 | DSGEGDFXAEGGGVR* | known | Fibrinogen cleavage peptide | Peptide | 1271 | 1.195 | 0.085 |  | 81 | 32.860 | 0.591 | 0 |  | 95 | 35.759 | 0.487 | 0 |  | 0.007737324 |
| M01572 | ergothioneine | known | Food component/Plant | Xenobiotics | 370 | 1.170 | 0.113 |  | 19 | 0.969 | 0.157 | 0.409169295 |  | 27 | 1.146 | 0.136 | 0.466840439 |  | 0.163136027 |
| M00527 | homostachydrine* | known | Food component/Plant | Xenobiotics | 1230 | 1.110 | 0.103 |  | 75 | 1.268 | 0.066 | 0.176808014 |  | 91 | 1.280 | 0.059 | 0.001133835 |  | 0.317439416 |
| M00599 | N-(2-furoyl)glycine | known | Food component/Plant | Xenobiotics | 43 | 1.039 | 0.207 |  | 0 | NA | NA | NA |  | 4 | 1.389 | 0.212 | 0.033216974 |  | 0.503058969 |
| M00575 | piperine | known | Food component/Plant | Xenobiotics | 1258 | 1.403 | 0.161 |  | 79 | 0.828 | 0.159 | 0.003409741 |  | 94 | 1.336 | 0.147 | 0.89084888 |  | 1.04E-06 |
| M15964 | quinate | known | Food component/Plant | Xenobiotics | 1163 | 1.422 | 0.125 |  | 80 | 0.944 | 0.172 | 0.00737218 |  | 90 | 1.786 | 0.164 | 0.069410124 |  | 0.030417697 |
| M35854 | saccharin | known | Food component/Plant | Xenobiotics | 499 | 1.827 | 0.209 |  | 39 | 1.889 | 0.591 | 0.630003852 |  | 33 | 2.098 | 0.621 | 0.412403138 |  | 6.07E-05 |
| M01640 | stachydrine | known | Food component/Plant | Xenobiotics | 1272 | 1.452 | 0.325 |  | 81 | 1.499 | 0.183 | 0.888338784 |  | 95 | 2.567 | 0.181 | 6.00E-09 |  | 8.28E-05 |
| M27738 | thymol sulfate | known | Food component/Plant | Xenobiotics | 330 | 1.655 | 0.190 |  | 6 | 2.005 | 1.039 | 0.762903722 |  | 33 | 3.962 | 0.562 | 3.38E-05 |  | 0.819979332 |
| M32586 | erythrose | known | Fructose, mannose, galactose, starch, and sucrose metabolism | Carbohydrate | 1266 | 1.172 | 0.270 |  | 81 | 1.145 | 0.103 | 0.907925471 |  | 95 | 1.264 | 0.102 | 0.467242886 |  | 0.635987182 |
| M34106 | fructose | known | Fructose, mannose, galactose, starch, and sucrose metabolism | Carbohydrate | 1272 | 1.192 | 0.253 |  | 81 | 1.436 | 0.111 | 0.039762536 |  | 95 | 1.262 | 0.099 | 0.458579085 |  | 0.479269818 |
| M27716 | mannitol | known | Fructose, mannose, galactose, starch, and sucrose metabolism | Carbohydrate | 1225 | 2.425 | 0.312 |  | 80 | 5.834 | 0.800 | 0.002016442 |  | 95 | 3.643 | 0.699 | 0.031830199 |  | 0.870145524 |
| M32593 | mannose | known | Fructose, mannose, galactose, starch, and sucrose metabolism | Carbohydrate | 1272 | 1.062 | 0.184 |  | 81 | 1.201 | 0.046 | 0.016651196 |  | 95 | 1.060 | 0.041 | 0.46943648 |  | 0.132873067 |
| M02137 | gamma-glutamylglutamine | known | gamma-glutamyl | Peptide | 1271 | 1.021 | 0.184 |  | 81 | 1.006 | 0.029 | 0.387433721 |  | 95 | 0.951 | 0.027 | 0.06853189 |  | 0.479269818 |
| M32401 | gamma-glutamylisoleucine* | known | gamma-glutamyl | Peptide | 1258 | 1.075 | 0.267 |  | 76 | 0.955 | 0.041 | 0.000643747 |  | 93 | 0.908 | 0.037 | 0.004301665 |  | 0.099552877 |
| M01508 | gamma-glutamylleucine | known | gamma-glutamyl | Peptide | 1272 | 1.083 | 0.147 |  | 81 | 0.831 | 0.037 | 4.04E-12 |  | 95 | 0.822 | 0.033 | 6.64E-09 |  | 0.867848946 |
| M01827 | gamma-glutamylphenylalanine | known | gamma-glutamyl | Peptide | 1272 | 1.065 | 0.147 |  | 81 | 0.962 | 0.037 | 0.001512284 |  | 95 | 0.895 | 0.034 | 0.000113379 |  | 0.190700309 |
| M01561 | gamma-glutamylthreonine* | known | gamma-glutamyl | Peptide | 1261 | 1.054 | 0.127 |  | 79 | 1.103 | 0.037 | 0.216117671 |  | 94 | 0.909 | 0.033 | 0.000210444 |  | 0.276859678 |
| M33420 | gamma-glutamyltyrosine | known | gamma-glutamyl | Peptide | 1271 | 1.049 | 0.218 |  | 81 | 1.099 | 0.037 | 0.638812043 |  | 94 | 0.917 | 0.033 | 0.004933486 |  | 6.54E-05 |
| M31555 | gamma-glutamylvaline | known | gamma-glutamyl | Peptide | 1272 | 1.076 | 0.187 |  | 81 | 0.813 | 0.035 | 5.94E-16 |  | 95 | 0.802 | 0.032 | 2.59E-11 |  | 0.025192827 |
| M33453 | glutamate | known | Glutamate metabolism | Amino acid | 1272 | 1.130 | 0.181 |  | 81 | 0.645 | 0.056 | 2.39E-17 |  | 95 | 0.585 | 0.050 | 6.35E-23 |  | 0.01878201 |
| M01564 | glutamine | known | Glutamate metabolism | Amino acid | 1271 | 0.990 | 0.119 |  | 81 | 1.162 | 0.015 | 4.95E-27 |  | 95 | 1.131 | 0.014 | 6.30E-24 |  | 0.299466671 |
| M01303 | pyroglutamine* | known | Glutamate metabolism | Amino acid | 1272 | 1.192 | 0.152 |  | 81 | 1.838 | 0.083 | 2.86E-10 |  | 95 | 1.285 | 0.074 | 9.99E-05 |  | 5.98E-12 |
| M37058 | 5-oxoproline | known | Glutathione metabolism | Amino acid | 1272 | 1.040 | 0.137 |  | 81 | 0.837 | 0.021 | 2.44E-23 |  | 95 | 0.721 | 0.019 | 1.40E-55 |  | 0.000254077 |
| M15488 | choline | known | Glycerolipid metabolism | Lipid | 1272 | 1.027 | 0.076 |  | 81 | 0.736 | 0.017 | 2.45E-65 |  | 95 | 0.715 | 0.015 | 1.11E-76 |  | 0.412270502 |
| M11438 | glycerol | known | Glycerolipid metabolism | Lipid | 1272 | 1.097 | 0.072 |  | 81 | 0.875 | 0.046 | 2.19E-09 |  | 95 | 0.923 | 0.041 | 2.62E-08 |  | 0.333453591 |
| M22842 | glycerol 3-phosphate (G3P) | known | Glycerolipid metabolism | Lipid | 1272 | 1.071 | 0.449 |  | 81 | 0.824 | 0.045 | 4.18E-07 |  | 95 | 0.854 | 0.041 | 1.05E-08 |  | 0.416341432 |
| M01114 | glycerophosphorylcholine (GPC) | known | Glycerolipid metabolism | Lipid | 1272 | 1.056 | 0.311 |  | 81 | 0.758 | 0.034 | 2.81E-15 |  | 95 | 0.733 | 0.030 | 1.15E-26 |  | 0.87056058 |
| M32346 | betaine | known | Glycine, serine and threonine metabolism | Amino acid | 1272 | 1.032 | 0.411 |  | 81 | 0.871 | 0.034 | 6.73E-06 |  | 95 | 1.007 | 0.031 | 0.524796544 |  | 0.001266457 |
| M18476 | glycine | known | Glycine, serine and threonine metabolism | Amino acid | 1272 | 1.050 | 0.312 |  | 81 | 1.266 | 0.035 | 1.33E-08 |  | 95 | 1.014 | 0.031 | 0.009494715 |  | 0.115324597 |
| M18477 | N-acetylglycine | known | Glycine, serine and threonine metabolism | Amino acid | 1241 | 1.188 | 0.380 |  | 81 | 2.044 | 0.097 | 2.57E-17 |  | 95 | 1.411 | 0.082 | 0.03819622 |  | 0.151384702 |
| M39379 | N-acetylthreonine | known | Glycine, serine and threonine metabolism | Amino acid | 1171 | 1.033 |  |  | 76 | 1.160 | 0.041 | 0.107591899 |  | 82 | 1.168 | 0.042 | 0.000125918 |  | 0.013690427 |
| M27531 | serine | known | Glycine, serine and threonine metabolism | Amino acid | 1272 | 1.040 | 0.342 |  | 81 | 0.915 | 0.025 | 1.02E-07 |  | 95 | 0.838 | 0.023 | 9.25E-21 |  | 0.000239704 |
| M18494 | threonine | known | Glycine, serine and threonine metabolism | Amino acid | 1210 | 1.013 | 0.348 |  | 73 | 1.224 | 0.029 | 1.03E-16 |  | 87 | 1.082 | 0.026 | 0.027125624 |  | 0.004469178 |
| M18497 | 1,5-anhydroglucitol (1,5-AG) | known | Glycolysis, gluconeogenesis, pyruvate metabolism | Carbohydrate | 1272 | 1.001 | 0.362 |  | 81 | 1.005 | 0.040 | 0.595558577 |  | 95 | 1.012 | 0.037 | 0.363149087 |  | 0.844125708 |
| M12261 | 1,6-anhydroglucose | known | Glycolysis, gluconeogenesis, pyruvate metabolism | Carbohydrate | 740 | 3.241 | 0.283 |  | 39 | 1.102 | 1.648 | 0.455908529 |  | 58 | 1.766 | 1.321 | 0.131014833 |  | 0.362563476 |
| M36850 | glucose | known | Glycolysis, gluconeogenesis, pyruvate metabolism | Carbohydrate | 1271 | 1.102 | 0.320 |  | 81 | 1.015 | 0.047 | 0.023018663 |  | 95 | 1.054 | 0.042 | 0.465258835 |  | 0.00185077 |
| M01605 | glycerate | known | Glycolysis, gluconeogenesis, pyruvate metabolism | Carbohydrate | 1272 | 1.076 | 0.267 |  | 81 | 0.424 | 0.037 | 3.49E-64 |  | 95 | 0.412 | 0.034 | 1.91E-83 |  | 0.188339929 |
| M35472 | lactate | known | Glycolysis, gluconeogenesis, pyruvate metabolism | Carbohydrate | 1272 | 1.046 | 0.231 |  | 81 | 1.215 | 0.037 | 2.44E-05 |  | 95 | 1.192 | 0.034 | 6.36E-06 |  | 0.580163971 |
| M32654 | pyruvate | known | Glycolysis, gluconeogenesis, pyruvate metabolism | Carbohydrate | 1272 | 1.092 | 0.134 |  | 81 | 1.297 | 0.068 | 0.010249299 |  | 95 | 1.512 | 0.063 | 2.17E-10 |  | 0.008047533 |
| M32198 | 4-acetamidobutanoate | known | Guanidino and acetamido metabolism | Amino acid | 1256 | 1.024 | 0.139 |  | 80 | 1.301 | 0.044 | 8.27E-06 |  | 95 | 1.160 | 0.041 | 4.57E-05 |  | 0.144971378 |
| M15500 | bilirubin (E,E)* | known | Hemoglobin and porphyrin metabolism | Cofactors and vitamins | 1268 | 1.290 | 0.060 |  | 81 | 0.923 | 0.117 | 0.002384381 |  | 95 | 0.859 | 0.105 | 0.000663176 |  | 0.637684836 |
| M38178 | bilirubin (E,Z or Z,E)* | known | Hemoglobin and porphyrin metabolism | Cofactors and vitamins | 1066 | 1.342 | 0.206 |  | 67 | 0.587 | 0.119 | 5.16E-11 |  | 76 | 0.544 | 0.109 | 1.36E-12 |  | 0.874671988 |
| M33941 | bilirubin (Z,Z) | known | Hemoglobin and porphyrin metabolism | Cofactors and vitamins | 1123 | 1.191 | 0.206 |  | 74 | 5.019 | 0.177 | 6.48E-92 |  | 92 | 5.776 | 0.173 | 3.61E-125 |  | 0.086426239 |
| M32328 | biliverdin | known | Hemoglobin and porphyrin metabolism | Cofactors and vitamins | 828 | 1.270 | 0.180 |  | 74 | 11.644 | 0.503 | 1.05E-73 |  | 87 | 1.495 | 0.122 | 0.005341845 |  | 3.18E-12 |
| M34534 | heme* | known | Hemoglobin and porphyrin metabolism | Cofactors and vitamins | 1231 | 1.226 | 0.227 |  | 78 | 0.420 | 0.077 | 2.16E-25 |  | 92 | 0.456 | 0.070 | 1.59E-25 |  | 0.002348468 |
| M33936 | 3-methylhistidine | known | Histidine metabolism | Amino acid | 1029 | 1.987 | 0.198 |  | 69 | 2.759 | 0.367 | 0.109693045 |  | 81 | 2.845 | 0.339 | 0.008811671 |  | 0.002650713 |
| M35160 | histidine | known | Histidine metabolism | Amino acid | 1272 | 1.011 | 0.182 |  | 81 | 0.941 | 0.019 | 0.016165059 |  | 95 | 1.021 | 0.017 | 0.843043287 |  | 0.752311413 |
| M22189 | chiro-inositol | known | Inositol metabolism | Lipid | 583 | 1.751 | 0.169 |  | 45 | 1.760 | 0.390 | 0.958796654 |  | 50 | 3.760 | 0.397 | 1.35E-06 |  | 0.024674851 |
| M34409 | myo-inositol | known | Inositol metabolism | Lipid | 1272 | 1.055 | 0.153 |  | 81 | 1.042 | 0.051 | 0.163355999 |  | 95 | 1.221 | 0.053 | 0.000250517 |  | 0.092794988 |
| M35718 | scyllo-inositol | known | Inositol metabolism | Lipid | 1238 | 1.115 | 0.142 |  | 80 | 1.050 | 0.075 | 0.18370189 |  | 92 | 1.639 | 0.099 | 3.96E-07 |  | 0.05931664 |
| M19323 | 3-hydroxybutyrate (BHBA) | known | Ketone bodies | Lipid | 1272 | 1.620 | 0.160 |  | 81 | 1.878 | 0.217 | 0.524863988 |  | 95 | 1.795 | 0.194 | 0.270851184 |  | 0.094279875 |
| M32504 | alpha-ketoglutarate | known | Krebs cycle | Energy | 1088 | 1.237 | 0.171 |  | 66 | 1.067 | 0.127 | 0.275913032 |  | 81 | 1.065 | 0.114 | 0.321748015 |  | 0.955963835 |
| M18467 | citrate | known | Krebs cycle | Energy | 1272 | 1.037 | 0.182 |  | 81 | 0.945 | 0.033 | 7.38E-06 |  | 95 | 1.088 | 0.031 | 0.086457129 |  | 0.000235413 |
| M01105 | malate | known | Krebs cycle | Energy | 1272 | 1.117 | 0.119 |  | 81 | 0.895 | 0.060 | 9.94E-07 |  | 95 | 0.861 | 0.054 | 1.56E-05 |  | 0.840749702 |
| M34035 | succinylcarnitine | known | Krebs cycle | Energy | 1269 | 1.051 | 0.172 |  | 81 | 1.118 | 0.039 | 0.706207788 |  | 94 | 1.072 | 0.036 | 0.198572056 |  | 0.385732603 |
| M34732 | 10-heptadecenoate (17:1n7) | known | Long chain fatty acid | Lipid | 1272 | 1.060 | 0.111 |  | 81 | 0.900 | 0.048 | 6.09E-05 |  | 95 | 0.957 | 0.044 | 0.001657352 |  | 0.154573341 |
| M32412 | 10-nonadecenoate (19:1n9) | known | Long chain fatty acid | Lipid | 1272 | 1.058 | 0.222 |  | 81 | 0.876 | 0.051 | 1.27E-05 |  | 95 | 0.952 | 0.046 | 0.005373426 |  | 0.04705409 |
| M32452 | adrenate (22:4n6) | known | Long chain fatty acid | Lipid | 1272 | 1.075 | 0.141 |  | 81 | 0.890 | 0.047 | 5.23E-05 |  | 95 | 0.968 | 0.042 | 0.017011849 |  | 0.079826392 |
| M32455 | arachidonate (20:4n6) | known | Long chain fatty acid | Lipid | 1272 | 1.042 | 0.190 |  | 81 | 0.836 | 0.025 | 1.37E-13 |  | 95 | 0.831 | 0.023 | 6.85E-19 |  | 0.611548688 |
| M32458 | dihomo-linoleate (20:2n6) | known | Long chain fatty acid | Lipid | 1272 | 1.088 | 0.163 |  | 81 | 0.705 | 0.053 | 2.07E-14 |  | 95 | 0.898 | 0.048 | 0.000133503 |  | 4.15E-05 |
| M38768 | eicosenoate (20:1n9 or 11) | known | Long chain fatty acid | Lipid | 1272 | 1.092 | 0.124 |  | 81 | 0.864 | 0.070 | 0.000249581 |  | 95 | 0.992 | 0.063 | 0.146783976 |  | 0.006777639 |
| M37253 | margarate (17:0) | known | Long chain fatty acid | Lipid | 1272 | 1.045 | 0.135 |  | 81 | 0.886 | 0.040 | 1.46E-06 |  | 95 | 0.973 | 0.036 | 0.01335884 |  | 0.296915333 |
| M31787 | myristate (14:0) | known | Long chain fatty acid | Lipid | 1272 | 1.048 | 0.438 |  | 81 | 0.965 | 0.039 | 0.00541453 |  | 95 | 1.006 | 0.035 | 0.037836377 |  | 0.001886806 |
| M32388 | myristoleate (14:1n5) | known | Long chain fatty acid | Lipid | 1272 | 1.111 | 0.157 |  | 81 | 1.049 | 0.064 | 0.203534508 |  | 95 | 1.083 | 0.057 | 0.071280577 |  | 0.21703507 |
| M35678 | nonadecanoate (19:0) | known | Long chain fatty acid | Lipid | 1269 | 1.043 | 0.199 |  | 81 | 0.948 | 0.036 | 0.004005498 |  | 95 | 1.002 | 0.032 | 0.136378192 |  | 1 |
| M36754 | oleate (18:1n9) | known | Long chain fatty acid | Lipid | 1272 | 1.008 | 0.179 |  | 81 | 0.894 | 0.034 | 5.95E-05 |  | 95 | 0.998 | 0.030 | 0.493523667 |  | 0.552978977 |
| M32398 | palmitate (16:0) | known | Long chain fatty acid | Lipid | 1272 | 1.012 | 0.091 |  | 81 | 0.877 | 0.028 | 6.70E-08 |  | 95 | 0.969 | 0.025 | 0.059808231 |  | 0.056101784 |
| M35669 | palmitoleate (16:1n7) | known | Long chain fatty acid | Lipid | 1272 | 1.062 | 0.204 |  | 81 | 0.925 | 0.054 | 0.004200616 |  | 95 | 1.015 | 0.048 | 0.089585687 |  | 0.002422802 |
| M36802 | pentadecanoate (15:0) | known | Long chain fatty acid | Lipid | 1257 | 1.034 | 0.114 |  | 81 | 1.006 | 0.039 | 0.116152032 |  | 92 | 1.045 | 0.036 | 0.444509463 |  | 0.0454186 |
| M35675 | stearate (18:0) | known | Long chain fatty acid | Lipid | 1272 | 1.021 | 0.129 |  | 81 | 0.882 | 0.027 | 3.99E-08 |  | 95 | 0.976 | 0.025 | 0.079318689 |  | 0.004826146 |
| M17945 | stearidonate (18:4n3) | known | Long chain fatty acid | Lipid | 1267 | 1.213 | 0.135 |  | 81 | 0.736 | 0.209 | 0.025212642 |  | 95 | 1.133 | 0.189 | 0.476730694 |  | 0.000193415 |
| M15506 | glutaroyl carnitine | known | Lysine metabolism | Amino acid | 1268 | 1.043 | 0.062 |  | 81 | 1.347 | 0.049 | 6.66E-08 |  | 94 | 1.140 | 0.042 | 0.001875355 |  | 0.208991974 |
| M15122 | lysine | known | Lysine metabolism | Amino acid | 1272 | 1.029 | 0.148 |  | 81 | 0.986 | 0.025 | 0.080215185 |  | 95 | 0.956 | 0.023 | 0.005991693 |  | 1.73E-05 |
| M15365 | pipecolate | known | Lysine metabolism | Amino acid | 1272 | 1.329 | 0.151 |  | 81 | 0.851 | 0.176 | 0.019835304 |  | 95 | 1.238 | 0.160 | 0.918737777 |  | 0.43507275 |
| M15990 | 1-arachidonoylglycerophosphocholine* | known | Lysolipid | Lipid | 1272 | 1.066 | 0.165 |  | 81 | 0.990 | 0.049 | 0.477155362 |  | 95 | 0.966 | 0.043 | 0.073507288 |  | 0.792009611 |
| M37112 | 1-arachidonoylglycerophosphoethanolamine* | known | Lysolipid | Lipid | 1272 | 1.052 | 0.399 |  | 81 | 1.175 | 0.042 | 0.008949842 |  | 95 | 1.095 | 0.038 | 0.127916527 |  | 0.171685428 |
| M01481 | 1-arachidonoylglycerophosphoinositol* | known | Lysolipid | Lipid | 1272 | 1.063 | 0.159 |  | 81 | 0.896 | 0.040 | 5.27E-06 |  | 95 | 1.029 | 0.036 | 0.538937126 |  | 0.001561489 |
| M19934 | 1-docosahexaenoylglycerophosphocholine* | linoleat | Lysolipid | Lipid | 1272 | 1.111 | 0.104 |  | 81 | 1.112 | 0.064 | 0.820047808 |  | 95 | 1.108 | 0.058 | 0.662107136 |  | 0.633510773 |
| M32379 | 1-eicosadienoylglycerophosphocholine* | known | Lysolipid | Lipid | 1267 | 1.117 | 0.204 |  | 79 | 0.985 | 0.064 | 0.07099218 |  | 93 | 0.944 | 0.059 | 0.013030944 |  | 0.627923348 |
| M00542 | 1-eicosatrienoylglycerophosphocholine* | known | Lysolipid | Lipid | 1272 | 1.081 | 0.354 |  | 81 | 1.074 | 0.057 | 0.713547737 |  | 95 | 1.119 | 0.051 | 0.317606777 |  | 0.714897388 |
| M33971 | 1-heptadecanoylglycerophosphocholine | known | Lysolipid | Lipid | 1272 | 1.178 | 0.159 |  | 81 | 0.866 | 0.075 | 4.11E-05 |  | 94 | 0.755 | 0.068 | 5.58E-10 |  | 0.306928698 |
| M33972 | 1-linoleoylglycerophosphocholine | known | Lysolipid | Lipid | 1272 | 1.028 | 0.171 |  | 81 | 1.022 | 0.041 | 0.658190581 |  | 95 | 1.089 | 0.037 | 0.02829579 |  | 0.227711722 |
| M32980 | 1-linoleoylglycerophosphoethanolamine* | known | Lysolipid | Lipid | 1272 | 1.090 | 0.167 |  | 81 | 1.120 | 0.057 | 0.937248638 |  | 95 | 1.130 | 0.052 | 0.28469188 |  | 0.914532716 |
| M01110 | 1-myristoylglycerophosphocholine | known | Lysolipid | Lipid | 1272 | 1.140 | 0.122 |  | 81 | 1.046 | 0.072 | 0.469291064 |  | 95 | 0.944 | 0.063 | 0.001364572 |  | 0.896066302 |
| M17805 | 1-oleoylglycerophosphocholine | known | Lysolipid | Lipid | 1272 | 1.083 | 0.166 |  | 81 | 1.058 | 0.053 | 0.8933976 |  | 95 | 1.016 | 0.048 | 0.338031911 |  | 0.555098166 |
| M33587 | 1-oleoylglycerophosphoethanolamine | known | Lysolipid | Lipid | 1272 | 1.128 | 0.184 |  | 81 | 1.288 | 0.076 | 0.015109596 |  | 95 | 1.130 | 0.068 | 0.713646113 |  | 0.167414092 |
| M01121 | 1-palmitoleoylglycerophosphocholine* | known | Lysolipid | Lipid | 1272 | 1.125 | 0.134 |  | 81 | 0.910 | 0.060 | 0.004408063 |  | 95 | 0.878 | 0.053 | 9.77E-06 |  | 0.939925126 |
| M01365 | 1-palmitoylglycerophosphocholine | known | Lysolipid | Lipid | 1272 | 1.044 | 0.136 |  | 81 | 0.897 | 0.033 | 3.48E-05 |  | 95 | 0.880 | 0.030 | 3.53E-07 |  | 0.835702805 |
| M32418 | 1-palmitoylglycerophosphoethanolamine | known | Lysolipid | Lipid | 1272 | 1.086 | 0.183 |  | 81 | 1.119 | 0.057 | 0.654867474 |  | 95 | 0.945 | 0.051 | 0.004040625 |  | 0.010934035 |
| M01356 | 1-palmitoylglycerophosphoinositol* | known | Lysolipid | Lipid | 1191 | 1.063 | 0.131 |  | 74 | 0.910 | 0.044 | 0.000194689 |  | 86 | 1.007 | 0.041 | 0.101268351 |  | 0.231230241 |
| M01359 | 1-palmitoylplasmenylethanolamine* | known | Lysolipid | Lipid | 1271 | 1.120 | 0.111 |  | 81 | 0.814 | 0.062 | 2.03E-06 |  | 95 | 0.895 | 0.056 | 6.29E-05 |  | 0.124241927 |
| M01336 | 1-stearoylglycerophosphocholine | known | Lysolipid | Lipid | 1272 | 1.096 | 0.094 |  | 81 | 0.799 | 0.046 | 7.30E-09 |  | 95 | 0.780 | 0.041 | 3.39E-13 |  | 0.734296141 |
| M33447 | 1-stearoylglycerophosphoethanolamine | known | Lysolipid | Lipid | 1083 | 1.135 | 0.184 |  | 69 | 0.873 | 0.078 | 0.002115629 |  | 82 | 0.886 | 0.069 | 0.000288504 |  | 0.808725147 |
| M01361 | 1-stearoylglycerophosphoinositol | known | Lysolipid | Lipid | 1262 | 1.091 | 0.133 |  | 81 | 0.961 | 0.056 | 0.001701531 |  | 93 | 1.029 | 0.051 | 0.129847022 |  | 0.222471391 |
| M01358 | 2-linoleoylglycerophosphocholine* | known | Lysolipid | Lipid | 1272 | 1.074 | 0.096 |  | 81 | 1.065 | 0.055 | 0.623757172 |  | 95 | 1.170 | 0.050 | 0.028873176 |  | 0.162898975 |
| M33969 | 2-oleoylglycerophosphocholine* | known | Lysolipid | Lipid | 1272 | 1.116 | 0.202 |  | 81 | 1.196 | 0.066 | 0.056651048 |  | 95 | 1.168 | 0.059 | 0.216340271 |  | 0.801241656 |
| M33228 | 2-palmitoylglycerophosphocholine* | known | Lysolipid | Lipid | 1272 | 1.137 | 0.174 |  | 81 | 0.821 | 0.056 | 4.22E-07 |  | 95 | 0.783 | 0.050 | 4.82E-11 |  | 0.932772907 |
| M35186 | 2-stearoylglycerophosphocholine* | known | Lysolipid | Lipid | 1272 | 1.178 | 0.147 |  | 81 | 0.655 | 0.064 | 2.57E-14 |  | 95 | 0.686 | 0.058 | 1.28E-16 |  | 0.185087361 |
| M34214 | 10-undecenoate (11:1n1) | known | Medium chain fatty acid | Lipid | 1272 | 1.039 | 0.146 |  | 81 | 1.108 | 0.048 | 0.27457923 |  | 95 | 1.261 | 0.044 | 2.93E-06 |  | 0.019318314 |
| M33822 | 5-dodecenoate (12:1n7) | known | Medium chain fatty acid | Lipid | 1267 | 1.146 | 0.190 |  | 81 | 1.104 | 0.084 | 0.510710727 |  | 95 | 1.120 | 0.074 | 0.260235047 |  | 0.301401524 |
| M33871 | caprate (10:0) | known | Medium chain fatty acid | Lipid | 1272 | 1.049 | 0.188 |  | 81 | 1.118 | 0.040 | 0.089200314 |  | 95 | 1.149 | 0.035 | 0.021393184 |  | 0.375742345 |
| M33821 | caproate (6:0) | known | Medium chain fatty acid | Lipid | 1272 | 1.021 | 0.184 |  | 81 | 0.975 | 0.032 | 0.266324474 |  | 95 | 1.030 | 0.028 | 0.958447938 |  | 0.193246936 |
| M33957 | caprylate (8:0) | known | Medium chain fatty acid | Lipid | 1272 | 1.036 | 0.171 |  | 81 | 1.124 | 0.037 | 0.006873266 |  | 95 | 1.182 | 0.031 | 1.67E-05 |  | 0.256022383 |
| M34419 | heptanoate (7:0) | known | Medium chain fatty acid | Lipid | 1272 | 1.003 | 0.136 |  | 81 | 1.074 | 0.027 | 0.00073255 |  | 95 | 1.215 | 0.025 | 8.73E-16 |  | 0.248844492 |
| M32635 | laurate (12:0) | known | Medium chain fatty acid | Lipid | 1272 | 1.035 | 0.171 |  | 81 | 1.114 | 0.033 | 0.070363897 |  | 95 | 1.124 | 0.028 | 0.012207187 |  | 0.913729196 |
| M35626 | pelargonate (9:0) | known | Medium chain fatty acid | Lipid | 1272 | 1.006 | 0.202 |  | 81 | 1.109 | 0.029 | 1.57E-05 |  | 95 | 1.260 | 0.027 | 3.88E-19 |  | 0.012064909 |
| M33960 | undecanoate (11:0) | known | Medium chain fatty acid | Lipid | 1272 | 1.009 | 0.147 |  | 81 | 1.016 | 0.020 | 0.428743636 |  | 95 | 1.068 | 0.018 | 0.010738985 |  | 0.004140188 |
| M35628 | 1-oleoylglycerol (1-monoolein) | known | Monoacylglycerol | Lipid | 1272 | 1.550 | 0.189 |  | 80 | 0.794 | 0.185 | 6.03E-05 |  | 92 | 0.448 | 0.167 | 1.20E-09 |  | 0.001495003 |
| M33230 | 1-palmitoylglycerol (1-monopalmitin) | known | Monoacylglycerol | Lipid | 1271 | 1.244 | 0.204 |  | 81 | 0.705 | 0.098 | 3.77E-07 |  | 94 | 0.699 | 0.089 | 2.94E-09 |  | 0.928364083 |
| M33955 | 1-stearoylglycerol (1-monostearin) | known | Monoacylglycerol | Lipid | 1266 | 1.089 | 0.109 |  | 81 | 0.859 | 0.047 | 8.71E-07 |  | 95 | 0.948 | 0.043 | 0.00051558 |  | 0.04229 |
| M35631 | arabinose | known | Nucleotide sugars, pentose metabolism | Carbohydrate | 809 | 1.230 | 0.153 |  | 80 | 1.342 | 0.114 | 0.596982188 |  | 90 | 1.319 | 0.107 | 0.31800311 |  | 0.577409787 |
| M35305 | threitol | known | Nucleotide sugars, pentose metabolism | Carbohydrate | 1265 | 1.190 | 0.187 |  | 81 | 1.457 | 0.098 | 0.108736663 |  | 94 | 1.370 | 0.090 | 0.023984536 |  | 0.031569431 |
| M39270 | acetylphosphate | known | Oxidative phosphorylation | Energy | 1272 | 1.008 |  |  | 81 | 1.003 | 0.025 | 0.67276853 |  | 95 | 1.054 | 0.022 | 0.248447612 |  | 0.131575439 |
| M33961 | phosphate | known | Oxidative phosphorylation | Energy | 1272 | 1.012 | 0.135 |  | 81 | 1.016 | 0.024 | 0.961367327 |  | 95 | 1.081 | 0.022 | 0.073274498 |  | 4.44E-05 |
| M34416 | pantothenate | known | Pantothenate and CoA metabolism | Cofactors and vitamins | 1271 | 1.123 | 0.165 |  | 80 | 1.095 | 0.076 | 0.31179518 |  | 95 | 1.116 | 0.067 | 0.978573185 |  | 0.551395994 |
| M19324 | 3-(3-hydroxyphenyl)propionate | known | Phenylalanine & tyrosine metabolism | Amino acid | 210 | 1.616 | 0.181 |  | 14 | 1.068 | 0.640 | 0.437223377 |  | 25 | 1.280 | 0.485 | 0.395155672 |  | 0.500806843 |
| M35257 | 3-(4-hydroxyphenyl)lactate | known | Phenylalanine & tyrosine metabolism | Amino acid | 1272 | 1.048 | 0.165 |  | 81 | 1.353 | 0.040 | 2.65E-11 |  | 95 | 1.079 | 0.035 | 0.004008241 |  | 0.002678579 |
| M36593 | 3-methoxytyrosine | known | Phenylalanine & tyrosine metabolism | Amino acid | 1262 | 1.417 | 0.180 |  | 81 | 222.394 | 5.301 | 1.04E-233 |  | 95 | 13.145 | 1.210 | 1.37E-21 |  | 1.60E-42 |
| M35254 | 3-phenylpropionate (hydrocinnamate) | known | Phenylalanine & tyrosine metabolism | Amino acid | 1139 | 1.287 | 0.160 |  | 61 | 1.086 | 0.157 | 0.340876412 |  | 79 | 1.471 | 0.137 | 0.32042398 |  | 0.173648705 |
| M35253 | p-cresol sulfate | known | Phenylalanine & tyrosine metabolism | Amino acid | 1272 | 1.172 | 0.155 |  | 81 | 2.039 | 0.114 | 3.05E-08 |  | 95 | 1.233 | 0.101 | 0.25139825 |  | 0.3054823 |
| M35255 | phenol sulfate | known | Phenylalanine & tyrosine metabolism | Amino acid | 1272 | 1.555 | 0.151 |  | 81 | 1.782 | 0.209 | 0.329894284 |  | 95 | 2.254 | 0.226 | 0.001161632 |  | 0.734614893 |
| M32497 | phenylacetate | known | Phenylalanine & tyrosine metabolism | Amino acid | 643 | 1.092 | 0.156 |  | 48 | 1.258 | 0.090 | 0.33871655 |  | 48 | 1.095 | 0.086 | 0.991062478 |  | 0.00559944 |
| M33968 | phenylacetylglutamine | known | Phenylalanine & tyrosine metabolism | Amino acid | 1272 | 1.166 | 0.210 |  | 81 | 1.881 | 0.098 | 8.65E-07 |  | 95 | 1.218 | 0.087 | 0.190773089 |  | 0.51730455 |
| M10642 | phenylalanine | known | Phenylalanine & tyrosine metabolism | Amino acid | 1272 | 1.036 | 0.084 |  | 81 | 0.822 | 0.019 | 4.10E-30 |  | 95 | 0.783 | 0.017 | 1.40E-42 |  | 0.005154679 |
| M32489 | phenyllactate (PLA) | known | Phenylalanine & tyrosine metabolism | Amino acid | 1185 | 1.061 | 0.117 |  | 80 | 1.500 | 0.048 | 1.68E-17 |  | 85 | 1.121 | 0.044 | 0.011631926 |  | 0.062908319 |
| M32492 | tyrosine | known | Phenylalanine & tyrosine metabolism | Amino acid | 1272 | 1.010 | 0.112 |  | 81 | 1.173 | 0.025 | 1.25E-08 |  | 95 | 0.995 | 0.022 | 0.911348177 |  | 0.000566406 |
| M01644 | bradykinin, des-arg(9) | known | Polypeptide | Peptide | 913 | 1.432 | 0.123 |  | 76 | 2.712 | 0.240 | 7.36E-09 |  | 91 | 3.474 | 0.227 | 1.21E-17 |  | 0.016260638 |
| M01645 | HWESASXX* | known | Polypeptide | Peptide | 1271 | 1.168 | 0.111 |  | 79 | 0.267 | 0.070 | 2.20E-40 |  | 93 | 0.246 | 0.064 | 7.70E-45 |  | 0.38028178 |
| M12035 | hypoxanthine | known | Purine metabolism, (hypo)xanthine/inosine containing | Nucleotide | 1141 | 1.095 | 0.106 |  | 70 | 1.137 | 0.061 | 0.597424264 |  | 82 | 0.919 | 0.054 | 0.000714573 |  | 0.691365424 |
| M12067 | xanthine | known | Purine metabolism, (hypo)xanthine/inosine containing | Nucleotide | 1261 | 1.102 | 0.081 |  | 80 | 0.920 | 0.052 | 0.00036305 |  | 94 | 0.844 | 0.045 | 9.45E-08 |  | 0.808559085 |
| M27447 | N1-methyladenosine | known | Purine metabolism, adenine containing | Nucleotide | 1271 | 0.997 | 0.221 |  | 81 | 1.211 | 0.017 | 2.01E-26 |  | 95 | 1.168 | 0.016 | 4.67E-29 |  | 0.980875898 |
| M21184 | 7-methylguanine | known | Purine metabolism, guanine containing | Nucleotide | 1256 | 1.032 | 0.250 |  | 81 | 1.260 | 0.042 | 1.33E-06 |  | 93 | 1.089 | 0.037 | 0.03497163 |  | 0.171616573 |
| M21127 | N2,N2-dimethylguanosine | known | Purine metabolism, guanine containing | Nucleotide | 1059 | 1.031 | 0.176 |  | 69 | 1.242 | 0.053 | 0.005494456 |  | 82 | 1.162 | 0.047 | 0.002173941 |  | 0.431739624 |
| M21188 | allantoin | known | Purine metabolism, urate metabolism | Nucleotide | 1235 | 1.113 | 0.151 |  | 74 | 0.910 | 0.073 | 0.000394247 |  | 88 | 0.788 | 0.066 | 5.86E-05 |  | 0.401386635 |
| M33443 | urate | known | Purine metabolism, urate metabolism | Nucleotide | 1272 | 1.017 | 0.133 |  | 81 | 0.996 | 0.023 | 0.398340359 |  | 95 | 1.079 | 0.021 | 9.12E-07 |  | 0.387865794 |
| M37506 | pseudouridine | known | Pyrimidine metabolism, uracil containing | Nucleotide | 1272 | 1.025 | 0.108 |  | 81 | 1.256 | 0.031 | 1.76E-07 |  | 95 | 1.141 | 0.030 | 2.88E-06 |  | 0.982626727 |
| M37202 | uridine | known | Pyrimidine metabolism, uracil containing | Nucleotide | 1272 | 0.990 | 0.365 |  | 81 | 0.965 | 0.020 | 0.571440307 |  | 95 | 1.086 | 0.018 | 5.22E-07 |  | 0.85818079 |
| M37203 | valerate | known | Short chain fatty acid | Lipid | 1172 | 1.065 | 0.237 |  | 75 | 0.807 | 0.043 | 7.47E-09 |  | 77 | 0.830 | 0.042 | 1.60E-08 |  | 0.106496916 |
| M37190 | palmitoyl sphingomyelin | known | Sphingolipid | Lipid | 1272 | 1.006 | 0.420 |  | 81 | 1.080 | 0.028 | 0.06913987 |  | 95 | 1.057 | 0.025 | 0.21816924 |  | 0.114952842 |
| M37198 | 4-androsten-3beta,17beta-diol disulfate 1* | known | Sterol/Steroid | Lipid | 1271 | 1.659 |  |  | 81 | 1.118 | 0.218 | 0.30217346 |  | 95 | 1.068 | 0.194 | 0.055836677 |  | 0.399427175 |
| M36776 | 4-androsten-3beta,17beta-diol disulfate 2* | known | Sterol/Steroid | Lipid | 1270 | 1.102 | 0.128 |  | 81 | 0.986 | 0.067 | 0.362117202 |  | 95 | 0.970 | 0.060 | 0.603376802 |  | 0.776817936 |
| M31591 | 5alpha-androstan-3beta,17beta-diol disulfate | known | Sterol/Steroid | Lipid | 1240 | 1.657 | 0.335 |  | 76 | 1.305 | 0.208 | 0.280251643 |  | 88 | 1.046 | 0.187 | 0.15483471 |  | 0.636352037 |
| M00063 | 5alpha-pregnan-3beta,20alpha-diol disulfate | known | Sterol/Steroid | Lipid | 1160 | 1.202 | 0.086 |  | 78 | 1.805 | 0.126 | 6.67E-06 |  | 87 | 1.440 | 0.118 | 0.022361677 |  | 0.054371455 |
| M01712 | 7-alpha-hydroxy-3-oxo-4-cholestenoate (7-Hoca) | known | Sterol/Steroid | Lipid | 1272 | 1.049 | 0.139 |  | 81 | 1.081 | 0.040 | 0.771890314 |  | 95 | 1.124 | 0.038 | 0.008735741 |  | 0.447243463 |
| M01769 | androsterone sulfate | known | Sterol/Steroid | Lipid | 1270 | 1.182 | 0.095 |  | 80 | 1.125 | 0.096 | 0.286984368 |  | 95 | 1.020 | 0.086 | 0.230490526 |  | 0.14648021 |
| M32425 | cholesterol | known | Sterol/Steroid | Lipid | 1272 | 0.997 | 0.283 |  | 81 | 1.034 | 0.024 | 0.153924678 |  | 95 | 1.124 | 0.022 | 4.73E-07 |  | 0.133821461 |
| M33973 | cortisol | known | Sterol/Steroid | Lipid | 1272 | 1.012 | 0.307 |  | 81 | 1.197 | 0.039 | 5.49E-06 |  | 95 | 1.006 | 0.036 | 0.815249671 |  | 0.000729059 |
| M18474 | cortisone | known | Sterol/Steroid | Lipid | 1265 | 0.990 | 0.229 |  | 81 | 1.058 | 0.025 | 0.000605155 |  | 95 | 1.008 | 0.023 | 0.389034442 |  | 0.031050398 |
| M33488 | dehydroisoandrosterone sulfate (DHEA-S) | known | Sterol/Steroid | Lipid | 1272 | 1.210 | 0.196 |  | 81 | 0.922 | 0.092 | 0.53894703 |  | 95 | 1.087 | 0.083 | 0.538383572 |  | 0.723170336 |
| M03127 | epiandrosterone sulfate | known | Sterol/Steroid | Lipid | 1266 | 1.207 | 0.121 |  | 79 | 1.098 | 0.100 | 0.523451291 |  | 93 | 1.043 | 0.087 | 0.537576893 |  | 0.845715796 |
| M01123 | estrone 3-sulfate | known | Sterol/Steroid | Lipid | 28 | 1.008 | 0.349 |  | NA | NA | NA | NA |  | 0 | NA | NA | NA |  | 1 |
| M03147 | lathosterol | known | Sterol/Steroid | Lipid | 1143 | 1.090 | 0.116 |  | 71 | 1.074 | 0.063 | 0.803578083 |  | 78 | 1.180 | 0.060 | 0.194220018 |  | 0.340508528 |
| M00555 | erythritol | known | Sugar, sugar substitute, starch | Xenobiotics | 1271 | 1.085 | 0.375 |  | 81 | 1.166 | 0.055 | 0.979128741 |  | 95 | 1.168 | 0.052 | 0.032175377 |  | 0.262506018 |
| M15650 | cotinine | known | Tobacco metabolite | Xenobiotics | 197 | 1.090 | 0.059 |  | 4 | 0.522 | 0.308 | 0.056592038 |  | 11 | 1.235 | 0.189 | 0.52688779 |  | 0.083193205 |
| M35114 | alpha-tocopherol | known | Tocopherol metabolism | Cofactors and vitamins | 1272 | 1.060 | 0.143 |  | 81 | 0.943 | 0.041 | 0.001224958 |  | 95 | 1.098 | 0.037 | 0.514251073 |  | 0.000917049 |
| M01573 | gamma-tocopherol | known | Tocopherol metabolism | Cofactors and vitamins | 1220 | 1.183 | 0.227 |  | 78 | 0.934 | 0.095 | 0.010731324 |  | 90 | 1.210 | 0.087 | 0.77572452 |  | 0.34189982 |
| M35137 | 3-indoxyl sulfate | known | Tryptophan metabolism | Amino acid | 1272 | 1.068 | 0.118 |  | 81 | 1.477 | 0.078 | 2.37E-06 |  | 95 | 1.157 | 0.070 | 0.058094922 |  | 0.160172583 |
| M01107 | C-glycosyltryptophan* | known | Tryptophan metabolism | Amino acid | 1268 | 1.032 | 0.199 |  | 81 | 1.396 | 0.048 | 2.19E-08 |  | 95 | 1.188 | 0.045 | 9.86E-05 |  | 0.020030154 |
| M01604 | indoleacetate | known | Tryptophan metabolism | Amino acid | 1269 | 1.261 | 0.090 |  | 79 | 0.989 | 0.101 | 0.003066607 |  | 94 | 1.027 | 0.090 | 0.053297511 |  | 0.622243537 |
| M33442 | indolelactate | known | Tryptophan metabolism | Amino acid | 1065 | 1.065 | 0.085 |  | 68 | 1.105 | 0.049 | 0.322074718 |  | 80 | 1.130 | 0.045 | 0.016763061 |  | 0.06352638 |
| M00606 | indolepropionate | known | Tryptophan metabolism | Amino acid | 1268 | 1.177 | 0.089 |  | 81 | 0.887 | 0.130 | 0.035164513 |  | 95 | 1.392 | 0.120 | 0.076359682 |  | 0.510362151 |
| M22175 | kynurenine | known | Tryptophan metabolism | Amino acid | 1268 | 1.024 | 0.271 |  | 81 | 1.125 | 0.030 | 0.039611237 |  | 95 | 1.108 | 0.027 | 8.68E-05 |  | 0.555848123 |
| M37104 | serotonin (5HT) | known | Tryptophan metabolism | Amino acid | 1264 | 1.095 | 0.240 |  | 74 | 0.660 | 0.071 | 1.80E-08 |  | 94 | 0.980 | 0.063 | 0.021403195 |  | 0.004262024 |
| M18357 | tryptophan | known | Tryptophan metabolism | Amino acid | 1272 | 1.001 | 0.205 |  | 81 | 0.940 | 0.017 | 0.018052134 |  | 95 | 1.028 | 0.016 | 0.007251361 |  | 0.355015492 |
| M36756 | tryptophan betaine | known | Tryptophan metabolism | Amino acid | 1166 | 1.964 | 0.202 |  | 75 | 1.490 | 0.367 | 0.674000205 |  | 94 | 2.632 | 0.328 | 0.034012834 |  | 0.005087668 |
| M38150 | arginine | known | Urea cycle, arginine-, proline-, metabolism | Amino acid | 1272 | 1.025 | 0.201 |  | 81 | 0.775 | 0.020 | 1.38E-31 |  | 95 | 0.771 | 0.019 | 1.29E-41 |  | 0.65626026 |
| M35127 | citrulline | known | Urea cycle, arginine-, proline-, metabolism | Amino acid | 1272 | 1.016 | 0.135 |  | 81 | 0.989 | 0.032 | 0.045156059 |  | 95 | 1.084 | 0.030 | 0.005583751 |  | 0.002469003 |
| M31522 | dimethylarginine (SDMA + ADMA) | known | Urea cycle, arginine-, proline-, metabolism | Amino acid | 1272 | 0.998 | 0.225 |  | 81 | 1.114 | 0.026 | 0.00078807 |  | 95 | 1.102 | 0.024 | 1.95E-06 |  | 0.496525066 |
| M33801 | homocitrulline | known | Urea cycle, arginine-, proline-, metabolism | Amino acid | 1155 | 1.191 | 0.208 |  | 75 | 1.219 | 0.095 | 0.408474163 |  | 83 | 1.348 | 0.094 | 0.081413501 |  | 0.009221541 |
| M33084 | N-acetylornithine | known | Urea cycle, arginine-, proline-, metabolism | Amino acid | 1270 | 1.109 | 0.158 |  | 81 | 1.013 | 0.068 | 0.087021057 |  | 95 | 1.191 | 0.062 | 0.153326482 |  | 0.001310919 |
| M31548 | ornithine | known | Urea cycle, arginine-, proline-, metabolism | Amino acid | 1272 | 1.009 | 0.237 |  | 81 | 1.235 | 0.027 | 1.01E-14 |  | 95 | 1.042 | 0.024 | 0.04416002 |  | 0.965735534 |
| M36738 | proline | known | Urea cycle, arginine-, proline-, metabolism | Amino acid | 1272 | 1.034 | 0.123 |  | 81 | 1.170 | 0.032 | 0.000224744 |  | 95 | 0.979 | 0.028 | 0.508461779 |  | 0.000771442 |
| M02730 | trans-4-hydroxyproline | known | Urea cycle, arginine-, proline-, metabolism | Amino acid | 1272 | 1.167 | 0.085 |  | 81 | 1.198 | 0.116 | 0.964961204 |  | 95 | 1.044 | 0.105 | 0.430740746 |  | 0.941206195 |
| M34456 | urea | known | Urea cycle, arginine-, proline-, metabolism | Amino acid | 1272 | 1.038 | 0.156 |  | 81 | 1.297 | 0.049 | 0.000152592 |  | 94 | 1.140 | 0.043 | 0.001627888 |  | 0.000546808 |
| M18369 | 2-hydroxyisobutyrate | known | Valine, leucine and isoleucine metabolism | Amino acid | 1271 | 1.102 | 0.104 |  | 81 | 1.228 | 0.089 | 0.387370805 |  | 95 | 1.580 | 0.115 | 7.66E-06 |  | 0.139096003 |
| M33363 | 2-methylbutyroylcarnitine | known | Valine, leucine and isoleucine metabolism | Amino acid | 1271 | 1.044 | 0.126 |  | 80 | 1.052 | 0.038 | 0.563742169 |  | 95 | 1.010 | 0.034 | 0.616915533 |  | 0.386447693 |
| M33422 | 3-methyl-2-oxobutyrate | known | Valine, leucine and isoleucine metabolism | Amino acid | 1272 | 1.026 | 0.106 |  | 81 | 0.950 | 0.027 | 0.025805424 |  | 95 | 1.046 | 0.024 | 0.118499076 |  | 0.003834136 |
| M33364 | 3-methyl-2-oxovalerate | known | Valine, leucine and isoleucine metabolism | Amino acid | 1271 | 1.034 | 0.125 |  | 81 | 0.973 | 0.032 | 0.332676413 |  | 95 | 1.016 | 0.029 | 0.270262989 |  | 0.085679866 |
| M02734 | 4-methyl-2-oxopentanoate | known | Valine, leucine and isoleucine metabolism | Amino acid | 1269 | 1.016 | 0.103 |  | 81 | 0.929 | 0.031 | 0.139509622 |  | 95 | 1.024 | 0.028 | 0.060324565 |  | 0.005874716 |
| M32393 | alpha-hydroxyisovalerate | known | Valine, leucine and isoleucine metabolism | Amino acid | 1272 | 1.280 | 0.122 |  | 81 | 1.344 | 0.150 | 0.422917575 |  | 95 | 1.135 | 0.137 | 0.82952055 |  | 0.004203238 |
| M34420 | beta-hydroxyisovalerate | known | Valine, leucine and isoleucine metabolism | Amino acid | 1267 | 1.024 | 0.369 |  | 81 | 1.073 | 0.034 | 0.259900304 |  | 95 | 1.091 | 0.031 | 0.000232511 |  | 0.159830003 |
| M32836 | hydroxyisovaleroyl carnitine | known | Valine, leucine and isoleucine metabolism | Amino acid | 890 | 1.098 | 0.217 |  | 54 | 1.088 | 0.081 | 0.812036551 |  | 70 | 0.958 | 0.071 | 0.088739949 |  | 0.694228528 |
| M18281 | isobutyrylcarnitine | known | Valine, leucine and isoleucine metabolism | Amino acid | 1271 | 1.090 | 0.722 |  | 81 | 1.320 | 0.062 | 0.001402706 |  | 95 | 1.112 | 0.054 | 0.447090978 |  | 4.16E-06 |
| M36848 | isoleucine | known | Valine, leucine and isoleucine metabolism | Amino acid | 1272 | 1.020 | 0.210 |  | 81 | 1.023 | 0.026 | 0.917066393 |  | 95 | 0.969 | 0.023 | 0.691687835 |  | 0.640522346 |
| M36099 | isovalerylcarnitine | known | Valine, leucine and isoleucine metabolism | Amino acid | 1272 | 1.075 | 0.359 |  | 81 | 0.975 | 0.046 | 0.128874462 |  | 95 | 0.890 | 0.042 | 0.00182593 |  | 0.004965838 |
| M35527 | leucine | known | Valine, leucine and isoleucine metabolism | Amino acid | 1272 | 1.019 | 0.205 |  | 81 | 0.929 | 0.021 | 3.27E-05 |  | 95 | 0.899 | 0.019 | 2.36E-06 |  | 0.666449453 |
| M36098 | levulinate (4-oxovalerate) | known | Valine, leucine and isoleucine metabolism | Amino acid | 1258 | 1.057 | 0.394 |  | 80 | 0.970 | 0.043 | 0.081250913 |  | 94 | 1.178 | 0.040 | 0.005004958 |  | 0.217635911 |
| M15778 | valine | known | Valine, leucine and isoleucine metabolism | Amino acid | 1272 | 1.011 | 0.118 |  | 81 | 0.976 | 0.018 | 0.084922038 |  | 95 | 0.965 | 0.016 | 0.130957618 |  | 0.293374743 |
| M35320 | pyridoxate | known | Vitamin B6 metabolism | Cofactors and vitamins | 1269 | 1.769 | 0.262 |  | 72 | 0.821 | 1.204 | 0.255072622 |  | 95 | 1.737 | 1.033 | 0.899548637 |  | 0.192108071 |
| M33173 | 1-methylurate | known | Xanthine metabolism | Xenobiotics | 1103 | 1.114 | 0.337 |  | 67 | 1.074 | 0.080 | 0.527046279 |  | 77 | 1.156 | 0.075 | 0.393259139 |  | 0.000751626 |
| M33178 | 1-methylxanthine | known | Xanthine metabolism | Xenobiotics | 941 | 1.188 | 0.107 |  | 56 | 0.922 | 0.103 | 0.03814026 |  | 74 | 1.225 | 0.090 | 0.627960939 |  | 0.36115986 |
| M15753 | 1,3,7-trimethylurate | known | Xanthine metabolism | Xenobiotics | 778 | 1.273 | 0.294 |  | 43 | 0.971 | 0.157 | 0.071154899 |  | 57 | 1.349 | 0.136 | 0.470075547 |  | 0.000428255 |
| M27728 | 1,7-dimethylurate | known | Xanthine metabolism | Xenobiotics | 1043 | 1.149 | 0.144 |  | 67 | 1.091 | 0.089 | 0.547002086 |  | 80 | 1.275 | 0.082 | 0.160707324 |  | 0.029236056 |
| M34365 | 3-methylxanthine | known | Xanthine metabolism | Xenobiotics | 906 | 1.167 | 0.167 |  | 53 | 1.121 | 0.111 | 0.392787101 |  | 75 | 1.429 | 0.094 | 0.002424694 |  | 0.242631244 |
| M12032 | 7-methylxanthine | known | Xanthine metabolism | Xenobiotics | 1043 | 1.163 |  |  | 62 | 1.330 | 0.094 | 0.2247083 |  | 84 | 1.525 | 0.084 | 8.40E-06 |  | 0.234147893 |
| M36097 | caffeine | known | Xanthine metabolism | Xenobiotics | 1223 | 1.804 | 0.621 |  | 77 | 1.350 | 0.276 | 0.436996974 |  | 90 | 1.898 | 0.253 | 0.907145864 |  | 0.0165744 |
| M38658 | paraxanthine | known | Xanthine metabolism | Xenobiotics | 1174 | 1.280 |  |  | 78 | 1.112 | 0.134 | 0.70097103 |  | 88 | 1.422 | 0.124 | 0.427902899 |  | 0.212153974 |
| M37033 | theobromine | known | Xanthine metabolism | Xenobiotics | 1251 | 1.284 | 0.004 |  | 79 | 1.761 | 0.145 | 0.003232545 |  | 94 | 1.984 | 0.123 | 3.08E-08 |  | 0.9024587 |
| M35322 | theophylline | known | Xanthine metabolism | Xenobiotics | 1150 | 1.862 | 0.171 |  | 74 | 1.175 | 0.806 | 0.249404909 |  | 87 | 1.273 | 0.725 | 0.467002615 |  | 0.05281765 |
| M33085 | X - 01911 | unknown |  |  | 1189 | 1.368 | 0.000 |  | 65 | 0.714 | 0.143 | 6.89E-05 |  | 92 | 1.184 | 0.122 | 0.445701874 |  | 0.000568912 |
| M17799 | X - 02249 | unknown |  |  | 1272 | 1.094 | 0.060 |  | 81 | 1.079 | 0.058 | 0.221758563 |  | 95 | 1.146 | 0.055 | 0.434010777 |  | 0.268161371 |
| M18037 | X - 02269 | unknown |  |  | 1267 | 1.216 | 0.229 |  | 80 | 1.028 | 0.107 | 0.063972906 |  | 95 | 1.360 | 0.099 | 0.100115055 |  | 0.00232184 |
| M34109 | X - 02973 | unknown |  |  | 1272 | 1.020 | 0.566 |  | 81 | 0.898 | 0.017 | 9.56E-12 |  | 95 | 0.900 | 0.016 | 1.29E-13 |  | 0.822135564 |
| M12122 | X - 03003 | unknown |  |  | 1268 | 1.034 | 0.000 |  | 81 | 1.244 | 0.041 | 1.56E-05 |  | 95 | 0.917 | 0.031 | 0.000466721 |  | 0.00084811 |
| M33423 | X - 03056 | unknown |  |  | 1270 | 1.083 | 0.532 |  | 80 | 0.638 | 0.046 | 1.22E-24 |  | 95 | 1.061 | 0.042 | 0.883214696 |  | 1.58E-13 |
| M33139 | X - 03088 | unknown |  |  | 1271 | 1.044 | 0.000 |  | 81 | 0.806 | 0.030 | 1.10E-16 |  | 95 | 0.818 | 0.027 | 1.23E-16 |  | 0.559931777 |
| M01515 | X - 03094 | unknown |  |  | 1271 | 1.038 | 0.660 |  | 81 | 0.905 | 0.033 | 4.49E-05 |  | 95 | 1.044 | 0.030 | 0.857031012 |  | 0.004306269 |
| M33384 | X - 04357 | unknown |  |  | 1271 | 1.132 | 0.343 |  | 81 | 1.325 | 0.071 | 0.011755242 |  | 95 | 1.166 | 0.064 | 0.71894422 |  | 0.01726498 |
| M37459 | X - 04494 | unknown |  |  | 573 | 1.043 | 0.230 |  | 47 | 1.178 | 0.060 | 0.099940639 |  | 40 | 1.091 | 0.062 | 0.361828663 |  | 0.689597996 |
| M33009 | X - 04495 | unknown |  |  | 1262 | 1.165 | 0.192 |  | 81 | 1.106 | 0.072 | 0.504916447 |  | 95 | 1.253 | 0.067 | 0.000880544 |  | 0.011614524 |
| M31536 | X - 04498 | unknown |  |  | 1270 | 1.106 | 0.239 |  | 81 | 1.062 | 0.058 | 0.073830862 |  | 95 | 1.106 | 0.058 | 0.576437168 |  | 0.141818781 |
| M33935 | X - 04499 | unknown |  |  | 1271 | 1.123 | 0.380 |  | 79 | 0.670 | 0.051 | 2.13E-21 |  | 92 | 0.642 | 0.047 | 8.09E-24 |  | 0.604769272 |
| M18335 | X - 04500 | unknown |  |  | 793 | 2.018 | 0.324 |  | 50 | 1.384 | 0.577 | 0.197062251 |  | 67 | 2.835 | 0.521 | 0.058707133 |  | 0.044919062 |
| M21151 | X - 05426 | unknown |  |  | 1261 | 1.442 | 0.271 |  | 77 | 0.765 | 0.182 | 8.64E-05 |  | 94 | 1.353 | 0.167 | 0.52572599 |  | 4.18E-06 |
| M34384 | X - 05907 | unknown |  |  | 1262 | 1.033 | 0.451 |  | 80 | 0.917 | 0.036 | 0.008543233 |  | 94 | 1.031 | 0.033 | 0.722391872 |  | 0.423044674 |
| M36095 | X - 06126 | unknown |  |  | 1271 | 1.445 | 0.485 |  | 81 | 1.252 | 0.175 | 0.100844891 |  | 93 | 1.242 | 0.160 | 0.302099978 |  | 0.189538494 |
| M20699 | X - 06226 | unknown |  |  | 1271 | 1.011 | 0.137 |  | 81 | 1.031 | 0.024 | 0.683305016 |  | 95 | 1.077 | 0.022 | 0.005958986 |  | 0.240840236 |
| M00553 | X - 06246 | unknown |  |  | 1271 | 1.028 | 0.349 |  | 81 | 1.007 | 0.037 | 0.369089888 |  | 94 | 1.103 | 0.036 | 0.034092787 |  | 0.316671115 |
| M34404 | X - 06267 | unknown |  |  | 1232 | 1.044 | 0.208 |  | 77 | 1.017 | 0.044 | 0.147659209 |  | 89 | 1.118 | 0.041 | 0.041922422 |  | 0.052607073 |
| M34400 | X - 06307 | unknown |  |  | 1240 | 1.052 | 0.209 |  | 81 | 1.441 | 0.062 | 1.80E-06 |  | 94 | 1.032 | 0.055 | 0.991751896 |  | 0.000268084 |
| M34395 | X - 06350 | unknown |  |  | 1152 | 1.036 | 0.197 |  | 66 | 0.971 | 0.045 | 0.213294756 |  | 84 | 1.105 | 0.040 | 0.247726205 |  | 0.066784663 |
| M34389 | X - 06351 | unknown |  |  | 1137 | 1.088 | 0.230 |  | 75 | 1.347 | 0.070 | 0.005268269 |  | 86 | 1.375 | 0.070 | 8.24E-06 |  | 0.2941738 |
| M32445 | X - 07765 | unknown |  |  | 242 | 1.250 | 0.253 |  | 10 | 3.799 | 0.669 | 6.89E-05 |  | 24 | 1.935 | 0.391 | 0.107884477 |  | 0.322859395 |
| M34390 | X - 08402 | unknown |  |  | 1271 | 1.020 | 0.269 |  | 81 | 1.132 | 0.039 | 0.017446162 |  | 95 | 1.144 | 0.036 | 0.008232192 |  | 0.760829038 |
| M00569 | X - 08766 | unknown |  |  | 1198 | 1.040 | 0.439 |  | 69 | 0.986 | 0.050 | 0.30163368 |  | 88 | 1.081 | 0.044 | 0.677004353 |  | 0.450303054 |
| M18254 | X - 08988 | unknown |  |  | 1270 | 1.028 | 0.369 |  | 81 | 1.159 | 0.029 | 9.55E-05 |  | 95 | 1.019 | 0.026 | 0.251518079 |  | 0.001643591 |
| M18392 | X - 09026 | unknown |  |  | 1258 | 1.042 | 0.352 |  | 81 | 0.961 | 0.032 | 0.069735896 |  | 93 | 1.018 | 0.029 | 0.667129669 |  | 0.49038157 |
| M18394 | X - 09108 | unknown |  |  | 1244 | 1.023 | 0.315 |  | 79 | 1.167 | 0.037 | 0.001407639 |  | 95 | 1.235 | 0.034 | 1.59E-09 |  | 0.388000631 |
| M32735 | X - 09706 | unknown |  |  | 1272 | 1.026 | 0.318 |  | 81 | 1.354 | 0.041 | 1.01E-11 |  | 95 | 1.267 | 0.038 | 2.00E-12 |  | 0.503454284 |
| M32587 | X - 09789 | unknown |  |  | 1201 | 1.244 | 0.193 |  | 77 | 1.099 | 0.106 | 0.153593197 |  | 93 | 1.524 | 0.099 | 0.001756602 |  | 0.001555942 |
| M32549 | X - 10346 | unknown |  |  | 307 | 1.388 | 0.327 |  | 18 | 1.569 | 0.326 | 0.546574414 |  | 32 | 1.497 | 0.238 | 0.633832478 |  | 0.481001 |
| M12593 | X - 10395 | unknown |  |  | 1259 | 1.043 | 0.076 |  | 80 | 0.884 | 0.037 | 0.000794663 |  | 93 | 1.026 | 0.035 | 0.413858308 |  | 0.076642699 |
| M12626 | X - 10429 | unknown |  |  | 1228 | 1.037 | 0.133 |  | 79 | 0.873 | 0.038 | 0.001798204 |  | 90 | 1.034 | 0.035 | 0.757100689 |  | 0.005467799 |
| M32709 | X - 10500 | identified |  |  | 1272 | 1.006 | 0.156 |  | 81 | 1.023 | 0.023 | 0.887287749 |  | 95 | 1.093 | 0.022 | 0.006075969 |  | 0.209963804 |
| M12768 | X - 10506 | unknown |  |  | 1272 | 1.010 | 0.154 |  | 81 | 1.096 | 0.029 | 0.014726244 |  | 95 | 1.094 | 0.027 | 0.000481414 |  | 0.351132575 |
| M12770 | X - 10510 | unknown |  |  | 1272 | 1.026 | 0.183 |  | 81 | 1.164 | 0.037 | 0.01381678 |  | 95 | 1.128 | 0.034 | 0.118332312 |  | 0.746551903 |
| M12774 | X - 10810 | unknown |  |  | 1264 | 1.008 | 0.114 |  | 81 | 1.857 | 0.051 | 1.86E-49 |  | 95 | 1.444 | 0.046 | 2.34E-23 |  | 0.001380257 |
| M16634 | X - 11204 | unknown |  |  | 1272 | 1.035 | 0.235 |  | 81 | 0.948 | 0.034 | 0.10504512 |  | 95 | 1.136 | 0.031 | 0.002251375 |  | 0.001171027 |
| M16816 | X - 11247 | unknown |  |  | 1238 | 1.951 | 0.136 |  | 76 | 1.218 | 1.011 | 0.519546873 |  | 92 | 2.323 | 0.904 | 0.633852745 |  | 0.001098869 |
| M16818 | X - 11261 | unknown |  |  | 1272 | 1.214 | 0.203 |  | 81 | 1.202 | 0.109 | 0.671426739 |  | 95 | 1.708 | 0.107 | 5.27E-06 |  | 0.059058964 |
| M16821 | X - 11299 | unknown |  |  | 1117 | 2.302 | 0.158 |  | 80 | 4.968 | 0.631 | 7.04E-06 |  | 92 | 6.583 | 0.645 | 1.01E-10 |  | 0.301476665 |
| M16822 | X - 11315 | identified |  |  | 1272 | 1.108 | 0.124 |  | 81 | 1.164 | 0.066 | 0.63238938 |  | 95 | 1.222 | 0.060 | 0.146458735 |  | 0.841722474 |
| M16823 | X - 11317 | unknown |  |  | 1272 | 1.027 | 0.355 |  | 81 | 0.975 | 0.028 | 0.146642147 |  | 95 | 1.127 | 0.026 | 0.001104737 |  | 0.01695536 |
| M17059 | X - 11327 | unknown |  |  | 1272 | 1.028 | 0.437 |  | 81 | 0.911 | 0.034 | 0.006612026 |  | 95 | 1.089 | 0.031 | 0.095341652 |  | 0.001625731 |
| M18283 | X - 11334 | unknown |  |  | 1263 | 1.198 | 0.229 |  | 80 | 0.919 | 0.107 | 0.001773013 |  | 93 | 1.209 | 0.098 | 0.336185531 |  | 1.08E-06 |
| M18929 | X - 11372 | unknown |  |  | 1270 | 1.049 | 0.125 |  | 81 | 1.125 | 0.054 | 0.026638806 |  | 95 | 1.260 | 0.050 | 7.71E-05 |  | 0.828844544 |
| M32557 | X - 11381 | unknown |  |  | 1272 | 1.061 | 0.393 |  | 81 | 0.794 | 0.033 | 7.58E-16 |  | 95 | 0.762 | 0.030 | 3.29E-24 |  | 0.386860431 |
| M19362 | X - 11412 | unknown |  |  | 1064 | 1.035 | 0.099 |  | 64 | 0.989 | 0.043 | 0.50515694 |  | 86 | 1.061 | 0.037 | 0.908818012 |  | 0.451227352 |
| M19363 | X - 11422 | unknown |  |  | 990 | 1.152 | 0.156 |  | 62 | 1.068 | 0.098 | 0.264193092 |  | 70 | 0.987 | 0.090 | 0.079429616 |  | 0.292685398 |
| M19364 | X - 11423 | unknown |  |  | 1272 | 1.037 | 0.140 |  | 81 | 1.069 | 0.042 | 0.924977699 |  | 95 | 1.089 | 0.038 | 0.051366976 |  | 0.157711902 |
| M19368 | X - 11437 | unknown |  |  | 921 | 1.759 | 0.141 |  | 61 | 2.492 | 0.326 | 0.037274147 |  | 80 | 3.107 | 0.277 | 1.31E-05 |  | 0.031720709 |
| M19396 | X - 11438 | unknown |  |  | 1269 | 1.102 | 0.187 |  | 81 | 1.170 | 0.066 | 0.804777798 |  | 95 | 1.237 | 0.060 | 0.012783614 |  | 0.089539451 |
| M19414 | X - 11440 | unknown |  |  | 1265 | 1.229 | 0.144 |  | 81 | 1.256 | 0.099 | 0.416032974 |  | 95 | 1.035 | 0.089 | 0.534921687 |  | 0.227307985 |
| M19415 | X - 11441 | unknown |  |  | 1103 | 1.217 | 0.160 |  | 41 | 0.556 | 0.154 | 7.62E-06 |  | 58 | 0.997 | 0.130 | 0.186285646 |  | 0.000733347 |
| M32560 | X - 11442 | unknown |  |  | 1147 | 1.177 | 0.272 |  | 50 | 0.575 | 0.095 | 1.13E-10 |  | 64 | 0.843 | 0.083 | 0.001299048 |  | 0.010132272 |
| M21630 | X - 11444 | unknown |  |  | 1264 | 1.083 | 0.143 |  | 81 | 1.257 | 0.062 | 0.04333446 |  | 95 | 1.144 | 0.061 | 0.038011863 |  | 0.564901211 |
| M22032 | X - 11452 | unknown |  |  | 1183 | 1.257 | 0.167 |  | 70 | 4.044 | 0.330 | 6.33E-17 |  | 89 | 1.179 | 0.113 | 0.954655052 |  | 0.096694549 |
| M22481 | X - 11469 | unknown |  |  | 1270 | 1.212 | 0.151 |  | 80 | 1.063 | 0.108 | 0.149475968 |  | 94 | 1.412 | 0.100 | 0.031711767 |  | 0.001599027 |
| M22548 | X - 11470 | unknown |  |  | 1246 | 1.103 | 0.136 |  | 80 | 1.110 | 0.064 | 0.754707383 |  | 94 | 1.304 | 0.073 | 0.000536921 |  | 0.548842616 |
| M22649 | X - 11478 | unknown |  |  | 1089 | 1.213 | 0.125 |  | 76 | 1.222 | 0.118 | 0.970662345 |  | 88 | 1.573 | 0.114 | 0.002537567 |  | 0.298713313 |
| M24074 | X - 11483 | unknown |  |  | 528 | 1.462 | 0.123 |  | 65 | 1.661 | 0.340 | 0.392309434 |  | 64 | 2.188 | 0.338 | 0.032266928 |  | 0.290496942 |
| M32753 | X - 11485 | unknown |  |  | 1007 | 1.182 | 0.293 |  | 46 | 0.914 | 0.103 | 0.036154259 |  | 59 | 1.123 | 0.091 | 0.779123452 |  | 0.066880995 |
| M33782 | X - 11491 | unknown |  |  | 1173 | 1.419 | 0.346 |  | 76 | 1.956 | 0.194 | 0.12941255 |  | 86 | 1.463 | 0.173 | 0.332322098 |  | 0.136622825 |
| M25459 | X - 11497 | unknown |  |  | 1272 | 0.996 | 0.125 |  | 81 | 1.020 | 0.031 | 0.299912191 |  | 95 | 1.120 | 0.028 | 3.98E-05 |  | 0.108003064 |
| M25599 | X - 11521 | unknown |  |  | 1240 | 1.174 | 0.156 |  | 79 | 1.079 | 0.089 | 0.103256562 |  | 94 | 1.263 | 0.082 | 0.149190281 |  | 0.021655259 |
| M27256 | X - 11529 | unknown |  |  | 1245 | 1.676 | 0.095 |  | 79 | 2.270 | 0.271 | 0.035796114 |  | 90 | 1.144 | 0.238 | 0.121330724 |  | 0.001851706 |
| M27273 | X - 11530 | unknown |  |  | 1260 | 1.175 | 0.118 |  | 78 | 0.660 | 0.081 | 1.41E-09 |  | 93 | 0.902 | 0.074 | 0.003629075 |  | 0.004165979 |
| M27278 | X - 11538 | unknown |  |  | 1271 | 1.155 | 0.149 |  | 80 | 1.515 | 0.085 | 0.000707553 |  | 95 | 1.239 | 0.073 | 0.090528412 |  | 0.16970797 |
| M28354 | X - 11550 | unknown |  |  | 1272 | 1.020 | 0.142 |  | 81 | 0.955 | 0.024 | 0.02840567 |  | 95 | 1.042 | 0.022 | 0.512471415 |  | 0.061817576 |
| M30805 | X - 11593 | unknown |  |  | 1272 | 1.042 | 0.190 |  | 80 | 0.850 | 0.034 | 2.95E-11 |  | 95 | 1.080 | 0.032 | 0.085849551 |  | 2.69E-07 |
| M32518 | X - 11787 | unknown |  |  | 1272 | 1.021 | 0.115 |  | 81 | 1.011 | 0.025 | 0.15572762 |  | 95 | 1.024 | 0.022 | 0.205903033 |  | 0.014422447 |
| M32564 | X - 11792 | unknown |  |  | 1271 | 1.086 | 0.409 |  | 80 | 0.146 | 0.043 | 3.78E-85 |  | 90 | 0.141 | 0.040 | 1.64E-112 |  | 0.014422963 |
| M32578 | X - 11793 | unknown |  |  | 1272 | 1.165 | 0.274 |  | 78 | 0.305 | 0.063 | 2.35E-40 |  | 91 | 0.298 | 0.057 | 1.57E-44 |  | 0.962734635 |
| M32616 | X - 11795 | unknown |  |  | 1271 | 1.088 | 0.451 |  | 81 | 1.110 | 0.053 | 0.735023533 |  | 94 | 1.023 | 0.048 | 0.711082421 |  | 0.689256821 |
| M32632 | X - 11799 | unknown |  |  | 981 | 2.352 | 0.178 |  | 40 | 0.564 | 0.624 | 0.022904038 |  | 59 | 2.158 | 0.507 | 0.709076212 |  | 7.74E-10 |
| M32634 | X - 11818 | unknown |  |  | 1261 | 1.064 | 0.113 |  | 80 | 1.209 | 0.049 | 0.103669414 |  | 94 | 1.047 | 0.044 | 0.566367273 |  | 0.553302914 |
| M32644 | X - 11820 | unknown |  |  | 1267 | 1.092 | 0.126 |  | 81 | 1.153 | 0.064 | 0.145966262 |  | 94 | 1.187 | 0.058 | 0.096083166 |  | 0.359078135 |
| M32651 | X - 11838 | unknown |  |  | 51 | 2.003 | 0.178 |  | 2 | 1.000 | 4.017 | 0.725925231 |  | 4 | 1.009 | 2.869 | 0.511107908 |  | 0.906622381 |
| M32689 | X - 11843 | unknown |  |  | 699 | 1.547 |  |  | 46 | 2.466 | 0.255 | 0.011332071 |  | 39 | 1.058 | 0.263 | 0.136371722 |  | 0.000968559 |
| M32691 | X - 11845 | unknown |  |  | 382 | 1.244 | 0.244 |  | 33 | 1.104 | 0.154 | 0.299193278 |  | 42 | 1.189 | 0.137 | 0.828861581 |  | 0.265504201 |
| M32698 | X - 11847 | unknown |  |  | 1066 | 1.549 | 0.107 |  | 72 | 1.719 | 0.230 | 0.517262726 |  | 84 | 1.999 | 0.218 | 0.031368965 |  | 0.866835169 |
| M32729 | X - 11849 | unknown |  |  | 850 | 1.735 | 0.135 |  | 53 | 1.593 | 0.354 | 0.502410463 |  | 77 | 1.812 | 0.286 | 0.812738991 |  | 0.423946981 |
| M32739 | X - 11850 | identified |  |  | 790 | 1.879 | 0.118 |  | 53 | 3.267 | 0.385 | 0.017414083 |  | 48 | 0.943 | 0.359 | 0.025574956 |  | 0.009119494 |
| M32740 | X - 11852 | identified |  |  | 860 | 2.724 | 0.098 |  | 53 | 2.298 | 0.660 | 0.510181096 |  | 53 | 5.204 | 0.888 | 0.002789635 |  | 0.109947247 |
| M32754 | X - 11858 | unknown |  |  | 451 | 1.872 | 0.479 |  | 32 | 1.336 | 0.854 | 0.620152817 |  | 46 | 1.836 | 0.716 | 0.924602613 |  | 0.189644563 |
| M32755 | X - 11876 | unknown |  |  | 1022 | 1.837 | 0.177 |  | 64 | 1.109 | 0.369 | 0.017715203 |  | 80 | 1.306 | 0.324 | 0.109362331 |  | 0.002409761 |
| M32757 | X - 11905 | unknown |  |  | 1000 | 1.172 | 0.281 |  | 63 | 1.069 | 0.095 | 0.077726285 |  | 76 | 1.058 | 0.084 | 0.287765307 |  | 0.946265332 |
| M32758 | X - 12007 | unknown |  |  | 276 | 1.357 | 0.223 |  | 21 | 1.026 | 0.317 | 0.020959123 |  | 19 | 1.074 | 0.305 | 0.144064016 |  | 0.268357368 |
| M32759 | X - 12013 | unknown |  |  | 463 | 1.344 | 0.220 |  | 35 | 1.630 | 0.186 | 0.65525813 |  | 20 | 1.089 | 0.230 | 0.838618242 |  | 0.659386318 |
| M32761 | X - 12029 | unknown |  |  | 756 | 1.199 | 0.171 |  | 50 | 1.063 | 0.257 | 0.623511107 |  | 50 | 1.239 | 0.251 | 0.97321143 |  | 0.172604896 |
| M32762 | X - 12038 | identified |  |  | 1272 | 1.019 | 0.288 |  | 80 | 1.004 | 0.025 | 0.89124698 |  | 95 | 1.058 | 0.022 | 0.155876057 |  | 0.643369342 |
| M32769 | X - 12039 | unknown |  |  | 1114 | 2.031 | 0.293 |  | 72 | 1.208 | 0.392 | 0.053128553 |  | 80 | 2.010 | 0.373 | 0.974595924 |  | 0.109912125 |
| M32786 | X - 12040 | unknown |  |  | 111 | 1.132 | 0.282 |  | 8 | 1.171 | 0.285 | 0.898619617 |  | 11 | 1.160 | 0.227 | 0.877128628 |  | 0.820617928 |
| M32787 | X - 12056 | unknown |  |  | 947 | 1.514 | 0.166 |  | 64 | 1.433 | 0.284 | 0.972970119 |  | 73 | 1.420 | 0.260 | 0.458058382 |  | 0.726248026 |
| M32795 | X - 12063 | unknown |  |  | 1214 | 1.219 | 0.251 |  | 71 | 1.014 | 0.096 | 0.019210122 |  | 90 | 1.079 | 0.084 | 0.135947371 |  | 0.215617399 |
| M32800 | X - 12092 | unknown |  |  | 1270 | 1.264 | 0.227 |  | 78 | 1.149 | 0.101 | 0.031822346 |  | 95 | 1.550 | 0.099 | 0.001343719 |  | 0.007785038 |
| M32802 | X - 12093 | unknown |  |  | 820 | 1.097 | 0.257 |  | 55 | 1.429 | 0.090 | 0.00066333 |  | 70 | 1.311 | 0.080 | 0.004991778 |  | 0.520418707 |
| M32808 | X - 12094 | unknown |  |  | 1250 | 1.102 | 0.271 |  | 75 | 0.932 | 0.068 | 0.006386068 |  | 93 | 1.272 | 0.063 | 0.005133897 |  | 8.85E-07 |
| M32814 | X - 12095 | unknown |  |  | 1271 | 1.093 | 0.097 |  | 79 | 0.914 | 0.057 | 0.000278831 |  | 94 | 1.167 | 0.053 | 0.1193483 |  | 5.35E-06 |
| M32838 | X - 12100 | unknown |  |  | 1272 | 1.022 | 0.209 |  | 81 | 1.201 | 0.038 | 0.001667821 |  | 95 | 1.198 | 0.038 | 5.05E-07 |  | 0.687423472 |
| M32846 | X - 12116 | unknown |  |  | 934 | 1.232 | 0.303 |  | 56 | 1.111 | 0.155 | 0.111340712 |  | 65 | 1.402 | 0.150 | 0.271033149 |  | 0.014777501 |
| M32847 | X - 12188 | unknown |  |  | 197 | 1.089 | 0.191 |  | 5 | 1.013 | 0.200 | 0.896898845 |  | 17 | 1.309 | 0.113 | 0.058386637 |  | 0.174900735 |
| M32854 | X - 12189 | unknown |  |  | 129 | 1.109 | 0.221 |  | 11 | 1.317 | 0.183 | 0.603889728 |  | 8 | 0.945 | 0.190 | 0.250686791 |  | 0.267369597 |
| M32855 | X - 12195 | unknown |  |  | 44 | 1.266 | 0.212 |  | 3 | 0.828 | 0.890 | 0.989247646 |  | 4 | 1.631 | 1.002 | 0.694791861 |  | 1 |
| M32857 | X - 12212 | unknown |  |  | 876 | 1.401 | 0.219 |  | 52 | 1.196 | 0.181 | 0.347759835 |  | 63 | 1.421 | 0.165 | 0.820357215 |  | 0.962546587 |
| M32863 | X - 12216 | unknown |  |  | 729 | 1.143 | 0.319 |  | 43 | 1.249 | 0.101 | 0.63180938 |  | 58 | 1.243 | 0.083 | 0.216597846 |  | 0.436103681 |
| M32867 | X - 12217 | unknown |  |  | 1180 | 1.203 | 0.046 |  | 75 | 1.004 | 0.124 | 0.056010848 |  | 91 | 1.470 | 0.119 | 0.009661484 |  | 0.00045766 |
| M32869 | X - 12230 | unknown |  |  | 1036 | 1.488 | 0.162 |  | 73 | 1.612 | 0.229 | 0.305079884 |  | 79 | 1.586 | 0.214 | 0.740777469 |  | 0.854083748 |
| M32910 | X - 12231 | identified |  |  | 1064 | 1.189 | 0.107 |  | 62 | 1.821 | 0.205 | 0.001371024 |  | 77 | 1.151 | 0.087 | 0.79326568 |  | 0.784742537 |
| M33131 | X - 12236 | identified |  |  | 460 | 1.423 | 0.181 |  | 29 | 0.931 | 0.261 | 0.04455338 |  | 44 | 1.288 | 0.209 | 0.45020614 |  | 0.013628811 |
| M33132 | X - 12244 | unknown |  |  | 1270 | 1.079 | 0.087 |  | 81 | 0.961 | 0.046 | 0.009351612 |  | 95 | 1.076 | 0.044 | 0.01610782 |  | 0.001709424 |
| M33137 | X - 12253 | unknown |  |  | 1115 | 1.380 | 0.230 |  | 69 | 0.811 | 0.139 | 0.000302229 |  | 83 | 1.356 | 0.128 | 0.430380726 |  | 6.38E-05 |
| M33138 | X - 12261 | identified |  |  | 297 | 1.093 | 0.200 |  | 24 | 1.991 | 0.139 | 1.35E-08 |  | 5 | 0.869 | 0.243 | 0.350302983 |  | 0.136655344 |
| M33140 | X - 12329 | unknown |  |  | 191 | 1.380 | 0.161 |  | 6 | 0.602 | 0.546 | 0.357880014 |  | 13 | 1.288 | 0.380 | 0.530298356 |  | 0.265205632 |
| M33144 | X - 12405 | unknown |  |  | 1169 | 1.116 | 0.351 |  | 74 | 1.457 | 0.124 | 0.019941325 |  | 85 | 1.179 | 0.112 | 0.338254338 |  | 0.07287443 |
| M33150 | X - 12407 | unknown |  |  | 723 | 1.958 | 0.307 |  | 39 | 0.953 | 0.500 | 0.008969093 |  | 53 | 1.056 | 0.414 | 0.054663454 |  | 0.495980243 |
| M33154 | X - 12442 | unknown |  |  | 1272 | 1.126 | 0.123 |  | 81 | 0.977 | 0.086 | 0.025989057 |  | 95 | 1.084 | 0.075 | 0.419033943 |  | 0.003875707 |
| M33163 | X - 12443 | unknown |  |  | 804 | 1.308 | 0.151 |  | 52 | 1.052 | 0.254 | 0.213376183 |  | 62 | 1.108 | 0.228 | 0.317902703 |  | 0.465066191 |
| M33165 | X - 12450 | unknown |  |  | 1272 | 1.050 | 0.241 |  | 81 | 0.966 | 0.028 | 0.001721006 |  | 95 | 0.931 | 0.026 | 1.46E-07 |  | 0.238195917 |
| M33183 | X - 12456 | unknown |  |  | 892 | 1.176 |  |  | 43 | 1.203 | 0.111 | 0.852240117 |  | 58 | 1.071 | 0.097 | 0.251963537 |  | 0.221565594 |
| M33188 | X - 12465 | unknown |  |  | 1242 | 1.216 | 0.442 |  | 79 | 1.233 | 0.097 | 0.567918883 |  | 94 | 1.313 | 0.091 | 0.069821582 |  | 0.319388327 |
| M33190 | X - 12510 | unknown |  |  | 1267 | 1.097 | 0.418 |  | 81 | 0.954 | 0.065 | 0.028333923 |  | 95 | 1.212 | 0.059 | 0.005623238 |  | 6.43E-05 |
| M33192 | X - 12524 | unknown |  |  | 1272 | 1.009 | 0.547 |  | 81 | 1.005 | 0.017 | 0.936061223 |  | 95 | 1.026 | 0.014 | 0.299040697 |  | 0.230632717 |
| M33194 | X - 12544 | unknown |  |  | 1009 | 2.695 | 0.527 |  | 59 | 1.889 | 0.762 | 0.71581004 |  | 69 | 2.129 | 0.695 | 0.698101927 |  | 0.780299535 |
| M33195 | X - 12556 | unknown |  |  | 1270 | 1.003 | 0.563 |  | 81 | 0.916 | 0.034 | 0.008166107 |  | 95 | 1.073 | 0.031 | 0.014128244 |  | 0.000802857 |
| M33197 | X - 12627 | unknown |  |  | 1184 | 1.231 | 0.516 |  | 69 | 0.779 | 0.105 | 1.14E-05 |  | 88 | 1.066 | 0.092 | 0.090874164 |  | 0.000206479 |
| M33203 | X - 12644 | unknown |  |  | 1272 | 1.036 | 0.437 |  | 81 | 1.177 | 0.044 | 0.029522334 |  | 95 | 1.144 | 0.040 | 0.00602442 |  | 0.592372645 |
| M33204 | X - 12645 | unknown |  |  | 1027 | 1.052 | 0.109 |  | 68 | 1.187 | 0.057 | 0.117850107 |  | 77 | 1.150 | 0.053 | 0.076106423 |  | 0.212815344 |
| M33221 | X - 12696 | unknown |  |  | 1223 | 0.996 | 0.243 |  | 80 | 1.451 | 0.043 | 1.66E-24 |  | 94 | 1.060 | 0.034 | 0.012707662 |  | 7.07E-05 |
| M33225 | X - 12704 | unknown |  |  | 528 | 1.492 | 0.210 |  | 25 | 1.288 | 0.625 | 0.635645139 |  | 42 | 1.402 | 0.474 | 0.909216321 |  | 0.387984543 |
| M33250 | X - 12712 | unknown |  |  | 82 | 1.367 | 0.160 |  | NA | NA | NA | NA |  | 3 | 1.170 | 1.359 | 0.699362855 |  | 1 |
| M33353 | X - 12719 | unknown |  |  | 208 | 1.227 | 0.166 |  | 12 | 1.011 | 0.261 | 0.386206885 |  | 26 | 1.097 | 0.179 | 0.454738181 |  | 0.903665204 |
| M33359 | X - 12726 | unknown |  |  | 1270 | 1.076 | 0.297 |  | 80 | 0.652 | 0.038 | 2.94E-28 |  | 94 | 0.624 | 0.034 | 8.76E-41 |  | 0.3527868 |
| M33380 | X - 12729 | unknown |  |  | 146 | 1.901 | 0.086 |  | 25 | 1.032 | 0.711 | 0.243104477 |  | 11 | 1.007 | 1.041 | 0.370503499 |  | 0.809994325 |
| M33389 | X - 12734 | unknown |  |  | 832 | 1.774 | 0.137 |  | 68 | 1.436 | 0.313 | 0.386752629 |  | 76 | 2.318 | 0.309 | 0.038757925 |  | 0.122763374 |
| M33390 | X - 12740 | unknown |  |  | 123 | 1.047 | 0.311 |  | 37 | 2.177 | 0.317 | 0.000430008 |  | 58 | 3.271 | 0.439 | 4.80E-08 |  | 0.243182155 |
| M33391 | X - 12749 | unknown |  |  | 1225 | 1.030 | 0.290 |  | 77 | 0.998 | 0.038 | 0.081633857 |  | 90 | 1.091 | 0.036 | 0.026429747 |  | 0.019495511 |
| M33408 | X - 12771 | unknown |  |  | 1218 | 1.064 | 0.309 |  | 79 | 1.207 | 0.060 | 0.000772728 |  | 88 | 1.039 | 0.054 | 0.739148124 |  | 0.090866183 |
| M33415 | X - 12776 | unknown |  |  | 1272 | 1.003 | 0.266 |  | 81 | 0.970 | 0.011 | 0.004838201 |  | 95 | 0.989 | 0.010 | 0.213062145 |  | 0.081914902 |
| M33507 | X - 12786 | unknown |  |  | 1232 | 1.058 | 0.271 |  | 79 | 1.543 | 0.065 | 1.06E-12 |  | 95 | 1.316 | 0.056 | 3.19E-05 |  | 0.016845853 |
| M33508 | X - 12798 | unknown |  |  | 1261 | 1.077 | 0.236 |  | 79 | 0.863 | 0.051 | 9.11E-06 |  | 94 | 0.970 | 0.045 | 0.011421382 |  | 0.009739776 |
| M33509 | X - 12816 | unknown |  |  | 985 | 1.293 | 0.179 |  | 46 | 0.371 | 0.159 | 2.37E-07 |  | 66 | 1.259 | 0.133 | 0.721547675 |  | 2.31E-12 |
| M33510 | X - 12830 | identified |  |  | 640 | 1.222 | 0.158 |  | 29 | 1.110 | 0.188 | 0.174045167 |  | 46 | 1.318 | 0.145 | 0.214084514 |  | 0.018290141 |
| M33515 | X - 12844 | identified |  |  | 1271 | 1.020 | 0.132 |  | 81 | 1.155 | 0.044 | 0.002615502 |  | 95 | 1.082 | 0.042 | 0.256800684 |  | 0.354628984 |
| M33531 | X - 12847 | unknown |  |  | 633 | 1.232 | 0.203 |  | 25 | 1.034 | 0.200 | 0.430019309 |  | 49 | 1.703 | 0.162 | 0.001783448 |  | 0.143847721 |
| M33609 | X - 12850 | unknown |  |  | 1144 | 1.423 | 0.241 |  | 71 | 2.904 | 0.422 | 0.00028246 |  | 84 | 1.112 | 0.209 | 0.372447785 |  | 0.07619136 |
| M33610 | X - 12851 | unknown |  |  | 965 | 2.069 | 0.186 |  | 59 | 5.844 | 0.609 | 2.72E-08 |  | 65 | 2.952 | 0.536 | 0.042198493 |  | 0.110830918 |
| M33616 | X - 12855 | unknown |  |  | 1207 | 1.055 |  |  | 74 | 1.110 | 0.050 | 0.672412625 |  | 92 | 1.181 | 0.046 | 0.001206722 |  | 0.081931592 |
| M33627 | X - 13069 | unknown |  |  | 1195 | 1.135 | 0.127 |  | 72 | 0.785 | 0.075 | 8.40E-07 |  | 89 | 1.079 | 0.067 | 0.109807113 |  | 0.00027855 |
| M33633 | X - 13215 | unknown |  |  | 1270 | 1.020 | 0.312 |  | 80 | 0.955 | 0.032 | 0.111900591 |  | 95 | 1.126 | 0.029 | 0.003320539 |  | 0.00238695 |
| M33637 | X - 13429 | unknown |  |  | 1073 | 1.426 | 0.236 |  | 73 | 2.188 | 0.211 | 0.00047478 |  | 73 | 1.144 | 0.194 | 0.305915574 |  | 0.020363209 |
| M33638 | X - 13431 | unknown |  |  | 1218 | 1.171 | 0.269 |  | 78 | 1.224 | 0.091 | 0.889855023 |  | 93 | 1.096 | 0.080 | 0.493638684 |  | 0.655272371 |
| M33652 | X - 13435 | unknown |  |  | 1212 | 1.083 | 0.259 |  | 76 | 0.912 | 0.054 | 2.77E-05 |  | 91 | 1.034 | 0.049 | 0.676183288 |  | 0.004289899 |
| M33653 | X - 13477 | unknown |  |  | 1245 | 1.045 | 0.259 |  | 78 | 0.867 | 0.036 | 3.47E-07 |  | 91 | 0.949 | 0.033 | 0.054026318 |  | 0.000734112 |
| M33658 | X - 13496 | unknown |  |  | 1272 | 1.001 | 0.276 |  | 81 | 0.997 | 0.021 | 0.954049746 |  | 95 | 1.084 | 0.020 | 0.000189411 |  | 0.062247987 |
| M33666 | X - 13548 | identified |  |  | 441 | 1.080 | 0.135 |  | 27 | 0.981 | 0.173 | 0.343741502 |  | 40 | 1.089 | 0.137 | 0.824853129 |  | 0.284539455 |
| M33675 | X - 13549 | unknown |  |  | 1194 | 1.025 | 0.331 |  | 72 | 1.026 | 0.036 | 0.666204466 |  | 88 | 1.052 | 0.032 | 0.450553711 |  | 0.969108419 |
| M33683 | X - 13553 | unknown |  |  | 334 | 1.035 | 0.278 |  | 10 | 1.130 | 0.115 | 0.662426935 |  | 25 | 1.113 | 0.074 | 0.457035413 |  | 0.943256932 |
| M33685 | X - 13619 | unknown |  |  | 1272 | 0.995 | 0.225 |  | 81 | 1.049 | 0.020 | 0.007130235 |  | 95 | 1.071 | 0.019 | 7.36E-05 |  | 0.546899361 |
| M33751 | X - 13658 | unknown |  |  | 761 | 1.752 | 0.320 |  | 19 | 1.039 | 0.691 | 0.237154995 |  | 29 | 0.991 | 0.554 | 0.167845186 |  | 0.857749174 |
| M33833 | X - 13671 | unknown |  |  | 1272 | 1.031 | 0.218 |  | 81 | 0.888 | 0.031 | 8.19E-06 |  | 95 | 0.923 | 0.027 | 6.29E-05 |  | 0.425801595 |
| M33835 | X - 13741 | unknown |  |  | 1125 | 1.669 | 0.236 |  | 65 | 0.757 | 0.239 | 0.000524176 |  | 83 | 1.453 | 0.210 | 0.397185448 |  | 6.27E-06 |
| M33864 | X - 13859 | unknown |  |  | 1194 | 1.057 | 0.212 |  | 72 | 0.983 | 0.044 | 0.04478578 |  | 87 | 0.929 | 0.039 | 0.00078357 |  | 0.849305736 |
| M33877 | X - 14056 | unknown |  |  | 1272 | 1.030 | 0.230 |  | 81 | 1.277 | 0.044 | 1.08E-08 |  | 95 | 1.302 | 0.041 | 1.04E-10 |  | 0.414254163 |
| M33883 | X - 14057 | identified |  |  | 1101 | 1.049 | 0.377 |  | 74 | 1.321 | 0.057 | 1.23E-06 |  | 85 | 1.517 | 0.055 | 2.70E-16 |  | 0.274299121 |
| M33884 | X - 14374 | identified |  |  | 1267 | 1.136 | 0.212 |  | 81 | 1.151 | 0.077 | 0.719329729 |  | 95 | 1.248 | 0.072 | 0.122108787 |  | 0.497580609 |
| M33885 | X - 14473 | unknown |  |  | 1232 | 1.153 | 0.368 |  | 74 | 1.002 | 0.081 | 0.267698326 |  | 90 | 1.151 | 0.073 | 0.427916016 |  | 0.035849833 |
| M33892 | X - 14588 | unknown |  |  | 1230 | 1.013 | 0.229 |  | 75 | 1.000 | 0.032 | 0.552021567 |  | 87 | 1.018 | 0.030 | 0.964629009 |  | 0.630162027 |
| M33901 | X - 14625 | unknown |  |  | 1202 | 1.051 | 0.229 |  | 70 | 0.941 | 0.038 | 0.001415146 |  | 79 | 0.803 | 0.035 | 1.10E-11 |  | 0.05970498 |
| M33910 | X - 14626 | unknown |  |  | 1244 | 1.080 | 0.210 |  | 80 | 1.322 | 0.052 | 4.02E-06 |  | 93 | 1.057 | 0.043 | 0.482676322 |  | 0.012432162 |
| M34040 | X - 14632 | identified |  |  | 216 | 1.039 | 0.207 |  | 11 | 1.180 | 0.108 | 0.38918751 |  | 22 | 1.059 | 0.078 | 0.769846852 |  | 0.226604449 |
| M34062 | X - 14658 | unknown |  |  | 606 | 1.529 | 0.090 |  | 35 | 5.326 | 0.958 | 2.08E-05 |  | 42 | 1.181 | 0.468 | 0.502274904 |  | 0.046721695 |
| M34102 | X - 14662 | unknown |  |  | 808 | 1.640 |  |  | 62 | 2.149 | 0.302 | 0.153905391 |  | 61 | 1.455 | 0.279 | 0.748015929 |  | 0.092057084 |
| M34112 | X - 14745 | unknown |  |  | 637 | 1.081 | 0.528 |  | 40 | 0.958 | 0.076 | 0.132179004 |  | 40 | 1.052 | 0.075 | 0.616132803 |  | 0.347362159 |
