# Supplementary material for: Alterations in Lipid and Inositol Metabolisms in Two Dopaminergic Disorders
Source: PLoS One. 2016 Jan 25;11(1):e0147129. doi: 10.1371/journal.pone.0147129 (PMC4726488; doi:10.1371/journal.pone.0147129)
Supplement: S2 Table — (DOCX) [file pone.0147129.s003.docx]

|  |  |  |  |  |  |  |
| --- | --- | --- | --- | --- | --- | --- |
| **Phenotype** | **Locus** | **Chr** | **Genomic position** | **Lead SNP** | **Risk allele** | **Ref** |
|  |  |  | **(hg19)** |  |  |  |
| PD | ***SNCA*** | 4 | 90678291 | rs2736990 | T | [^1^](#_ENREF_1) |
| PD | ***MAPT*** | 17 | 43718893 | rs393152 | G | [^1^](#_ENREF_1) |
| PD | ***PARK16/RAB7L1*** | 1 | 205752415 | rs947211 | A | [^2^](#_ENREF_2) |
| PD | ***BST1*** | 4 | 15737687 | rs4538475 | A | [^2^](#_ENREF_2) |
| PD | ***LRRK2*** | 12 | 40428311 | rs1994090 | T | [^2^](#_ENREF_2) |
| PD | ***HLA-DRA*** | 6 | 32517508 | rs3129882 | G | [^3^](#_ENREF_3) |
| PD | ***GAK*** | 4 | 848332 | rs11248051 | T | [^3^](#_ENREF_3) |
| PD | ***SYT11*** | 1 | 154105678 | not named | T | [^4^](#_ENREF_4) |
| PD | ***ACMSD*** | 2 | 135308851 | rs6710823 | A | [^4^](#_ENREF_4) |
| PD | ***STK39*** | 2 | 168825271 | rs2102808 | T | [^4^](#_ENREF_4) |
| PD | ***MCCC1/LAMP3*** | 3 | 184303969 | rs11711441 | G | [^4^](#_ENREF_4) |
| PD | ***CCDC62/HIP1R*** | 12 | 121862247 | rs12817488 | A | [^4^](#_ENREF_4) |
| PD | ***SCARB2*** | 4 | 77418010 | rs6812193 | T | [^5^](#_ENREF_5) |
| PD | ***SREBF/RAI*** | 17 | 17655826 | rs11868035 | A | [^5^](#_ENREF_5) |
| PD | ***STBD1*** | 4 | 77198736 | rs6812193 | T | [^6^](#_ENREF_6) |
| PD | ***GPNMB*** | 7 | 23305770 | rs156429 | G | [^6^](#_ENREF_6) |
| PD | ***FGF20*** | 8 | 16696841 | rs591323 | A | [^6^](#_ENREF_6) |
| PD | ***STX1B*** | 16 | 30981975 | rs4889603 | A | [^6^](#_ENREF_6) |
| PD | ***RIT2*** | 18 | 38927378 | rs12456492 | G | [^7^](#_ENREF_7) |
| PD | ***ITGA8*** | 10 | 15691549 | rs7077361 | T | [^8^](#_ENREF_8) |
| PD | ***DGKQ*** | 4 | 954359 | rs11248060 | T | [^8^](#_ENREF_8) |
| RLS | ***MEIS1*** | 2 | 66781453 | rs2300478 | G | [^9^](#_ENREF_9)^,^ [^10^](#_ENREF_10) |
| RLS | ***MEIS1*** | 2 | 66764308 | rs12469063 | G | [^9^](#_ENREF_9)^,^ [^10^](#_ENREF_10) |
| RLS | ***MEIS1*** | 2 | 66750564 | rs113851554 | T | [^11^](#_ENREF_11) |
| RLS | ***MEIS1*** | 2 | 66799986 | rs11693221 | T | [^12^](#_ENREF_12) |
| RLS | **intergenic** | 2 | 68070225 | rs6747972 | A | [^9^](#_ENREF_9) |
| RLS | ***TOX3/BC034767*** | 16 | 52624738 | rs3104767 | G | [^9^](#_ENREF_9) |
| RLS | ***BTBD9*** | 6 | 38365873 | rs9357271 | T | [^9^](#_ENREF_9)^,^ [^10^](#_ENREF_10) |
| RLS | ***BTBD9*** | 6 | 38440970 | rs3923809 | A | [^13^](#_ENREF_13) |
| RLS | ***PTPRD*** | 9 | 9261737 | rs4626664 | A | [^14^](#_ENREF_14) |
| RLS | ***PTPRD*** | 9 | 8846955 | rs1975197 | A | [^14^](#_ENREF_14) |
| RLS | ***MAP2K5/SKOR1*** | 15 | 68036852 | rs12593813 | G | [^9^](#_ENREF_9)^,^ [^10^](#_ENREF_10) |

REFERENCES

1. Simon-Sanchez J, Schulte C, Bras JM, et al. Genome-wide association study reveals genetic risk underlying Parkinson's disease. Nature genetics 2009;41:1308-1312.

2. Satake W, Nakabayashi Y, Mizuta I, et al. Genome-wide association study identifies common variants at four loci as genetic risk factors for Parkinson's disease. Nature genetics 2009;41:1303-1307.

3. Hamza TH, Zabetian CP, Tenesa A, et al. Common genetic variation in the HLA region is associated with late-onset sporadic Parkinson's disease. Nature genetics 2010;42:781-785.

4. International Parkinson Disease Genomics C, Nalls MA, Plagnol V, et al. Imputation of sequence variants for identification of genetic risks for Parkinson's disease: a meta-analysis of genome-wide association studies. Lancet 2011;377:641-649.

5. Do CB, Tung JY, Dorfman E, et al. Web-based genome-wide association study identifies two novel loci and a substantial genetic component for Parkinson's disease. PLoS genetics 2011;7:e1002141.

6. International Parkinson's Disease Genomics C, Wellcome Trust Case Control C. A two-stage meta-analysis identifies several new loci for Parkinson's disease. PLoS genetics 2011;7:e1002142.

7. Pankratz N, Beecham GW, DeStefano AL, et al. Meta-analysis of Parkinson's disease: identification of a novel locus, RIT2. Ann Neurol 2012;71:370-384.

8. Lill CM, Roehr JT, McQueen MB, et al. Comprehensive research synopsis and systematic meta-analyses in Parkinson's disease genetics: The PDGene database. PLoS genetics 2012;8:e1002548.

9. Schulte EC, Knauf F, Kemlink D, et al. Variant screening of the coding regions of MEIS1 in patients with restless legs syndrome. Neurology 2011;76:1106-1108.

10. Winkelmann J, Schormair B, Lichtner P, et al. Genome-wide association study of restless legs syndrome identifies common variants in three genomic regions. Nature genetics 2007;39:1000-1006.

11. Xiong L, Catoire H, Dion P, et al. MEIS1 intronic risk haplotype associated with restless legs syndrome affects its mRNA and protein expression levels. Human molecular genetics 2009;18:1065-1074.

12. Schulte EC, Kousi M, Tan PL, et al. Targeted Resequencing and Systematic In Vivo Functional Testing Identifies Rare Variants in MEIS1 as Significant Contributors to Restless Legs Syndrome. American journal of human genetics 2014;95:85-95.

13. Stefansson H, Rye DB, Hicks A, et al. A genetic risk factor for periodic limb movements in sleep. The New England journal of medicine 2007;357:639-647.

14. Schormair B, Kemlink D, Roeske D, et al. PTPRD (protein tyrosine phosphatase receptor type delta) is associated with restless legs syndrome. Nature genetics 2008;40:946-948.
